# Sodium-Permeable Ion Channels TRPM4 and TRPM5 are Functional in Human Gastric Parietal Cells in Culture and Modulate the Cellular Response to Bitter-Tasting Food Constituents

**DOI:** 10.1021/acs.jafc.3c09085

**Published:** 2024-02-20

**Authors:** Phil Richter, Gaby Andersen, Kristin Kahlenberg, Alina Ulrike Mueller, Philip Pirkwieser, Valerie Boger, Veronika Somoza

**Affiliations:** † TUM School of Life Sciences Weihenstephan, Technical University of Munich, Alte Akademie 8, Freising 85354, Germany; ‡ Leibniz Institute for Food Systems Biology at the Technical University of Munich, Lise-Meitner-Str. 34, Freising 85354, Germany; § Chair of Nutritional Systems Biology, TUM School of Life Sciences, Technical University of Munich, Lise-Meitner-Str. 34, Freising 85354, Germany; ∥ Department of Physiological Chemistry, Faculty of Chemistry, University of Vienna, Josef-Holaubek-Platz 2 (UZA II), Vienna 1090, Austria

**Keywords:** transient receptor potential channels (TRP) M4/M5, taste receptors, TAS2Rs, bitter taste signaling, sodium pathway

## Abstract

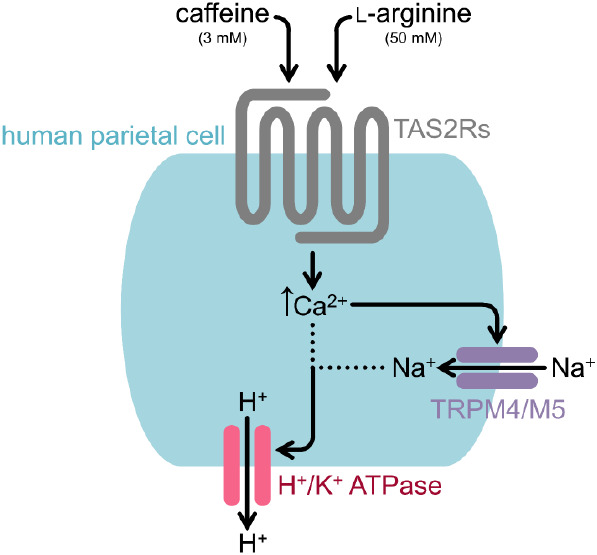

Gastric parietal cells secrete chloride ions and protons to form hydrochloric acid. Besides endogenous stimulants, e.g., acetylcholine, bitter-tasting food constituents, e.g., caffeine, induce proton secretion via interaction with bitter taste receptors (TAS2Rs), leading to increased cytosolic Ca^2+^ and cAMP concentrations. We hypothesized TAS2R activation by bitter tastants to result in proton secretion via cellular Na^+^ influx mediated by transient receptor potential channels (TRP) M4 and M5 in immortalized human parietal HGT-1 cells. Using the food-derived TAS2R agonists caffeine and l-arginine, we demonstrate both bitter compounds to induce a TRPM4/M5-mediated Na^+^ influx, with EC_50_ values of 0.65 and 10.38 mM, respectively, that stimulates cellular proton secretion. Functional involvement of TAS2Rs in the caffeine-evoked effect was demonstrated by means of the TAS2R antagonist homoeriodictyol, and stably CRISPR-Cas9-edited TAS2R43ko cells. Building on previous results, these data further support the suitability of HGT-1 cells as a surrogate cell model for taste cells. In addition, TRPM4/M5 mediated a Na^+^ influx after stimulating HGT-1 cells with the acetylcholine analogue carbachol, indicating an interaction of the digestion-associated cholinergic pathway with a taste-signaling pathway in parietal cells.

## Introduction

Parietal cells are highly specialized cells located within the gastric glands in the stomach. They are responsible for gastric acid production from the secretion of chloride ions and protons. Upon stimulation of the cells, H^+^/K^+^-ATPase-containing tubulovesicles are translocated to the apical membrane via exocytotic fusion, thereby enabling the secretion of protons into the stomach lumen. While gastric acid is essential for the digestion of dietary proteins and suppressing pathogens, any imbalance contributes to pathophysiological conditions like atrophic gastritis, peptic ulcers, gastroesophageal reflux disease, and vitamin B12 deficiency.^1^

*In vivo* gastric acid secretion is triggered by endocrine, paracrine, and neuronal signals. Two different intracellular signaling pathways have particularly been described to play a crucial role: the cAMP-mediated PKA activation pathway, which is induced by histaminergic stimulation of the H_2_ receptor, and the Ca^2+^ pathway, typically induced by binding of gastrin to the CCK_B_ receptor and cholinergic stimulation of the muscarinic M_3_ receptor.^2^ The latter positively couples to phospholipase C through G_q/11_, inducing inositol 1,4,5-trisphosphate (IP_3_) and diacylglycerol generation, followed by increased intracellular Ca^2+^.^3^ Yet, interactions between the two pathways may occur.^4^ With respect to food ingredients, the bitter-tasting compound caffeine was shown to induce gastric acid secretion via the activation of bitter taste receptors (TAS2Rs).^5^ Moreover, a positive correlation was shown between the perceived bitterness of caffeine and the extent of its ability to stimulate gastric acid secretion,^5^ implicating the activation of similar intracellular signaling pathways in taste and parietal cells by bitter compounds targeting TAS2Rs.

In taste cells located on the tongue, activation of TAS2Rs by bitter compounds initiates dissociation of the G protein α-gustducin from its β3γ13 subunits. The latter activates PLCβ2 (1-phosphatidylinositol-4,5-bisphosphate phosphodiesterase beta-2), leading to IP_3_ (inositol-1,4,5-trisphosphate) generation and a release of Ca^2+^ from IP_3_ sensitive Ca^2+^ stores, resulting in Na^+^ influx through the transient receptor potential cation channel subfamily M (M for melastatin) members 4 and 5 (TRPM4/5),^6−8^ subsequent ATP release, and finally activation of the gustatory cortex.^9,10^

The TRPM subfamily comprises eight members with diverse functional properties.^11^ TRPM4 and M5 are activated by increased cytosolic Ca^2+^ concentrations and are insofar exceptional since they are the only two TRP channels that are monovalent, cation-selective, and impermeable to divalent cations, e.g., Ca^2+^.^6,7,12,13^ In contrast, most TRPs are nonselective cation channels that are permeable to monovalent and divalent cations like Na^+^ and Ca^2+^, and despite the fact that TRPM5 is about 20-fold more sensitive to Ca^2+^ than TRPM4,^14^ both channels contribute about equally to taste perception.^8^ TRPM4 and TRPM5 were found to be expressed in the stomach, albeit the respective cell type was not identified.^15^ A more detailed analysis revealed the expression of TRPM5 in the principal part of the gastric glands, most abundantly in the cardiac region.^16^ The functional roles of TRPM4 and M5 in this tissue, however, have yet to be elucidated.

A well-established model for the analyses of mechanisms of gastric acid secretion and gastric cancer is the HGT-1 cell line, a human gastric cancer cell line that was established from a primary tumor of a 60-year-old male patient.^17^ HGT-1 cells do not secrete mucus but maintain expression of functional histamine H_2_ receptors and the principal transporters found in primary nontumor acid-secreting parietal cells.^18^ Additionally, these cells express functional taste receptors as well as PLCβ2, GNAT2 (G Protein Subunit Alpha Transducin 2), GNAT3 (Gustducin alpha-3 chain), and IP_3_Rs (IP_3_ receptors), some of the main components of the taste signaling pathway.^5,19^ Moreover, results from HGT-1 cells regarding proton secretion upon stimulation with bitter compounds were verified *in vivo*.^5^ Whether the HGT-1 cells also express the TRP channels M4 and M5, and if so, whether TRPM4 and TRPM5 play a role in the signaling process of food-derived bitter compounds, comparable to taste cells, is not known. This, however, would allow the conclusion that HGT-1 cells may serve as a suitable surrogate for investigating taste-active compounds and their ability to induce cellular and physiological responses, e.g., taste perception.

Yet, the available data suggest that HGT-1 cells express a fully functional bitter taste signaling pathway. It is, therefore, of great interest not only to investigate the impact of bitter compounds on cellular Ca^2+^ mobilization in response to TAS2R activation, but also to clarify the possible secondary effects resulting from activated signaling pathways, such as an activity of cellular ion channels. Thus, we hypothesized that the ion channels TRPM4 and TRPM5 are expressed in the parietal cell line HGT-1 and are functionally linked to TAS2R signaling pathways, thereby contributing to proton secretion induced by bitter compounds.

## Materials and Methods

### Chemicals

Na^+^ binding benzofuran isophthalate acetoxymethyl ester (SBFI-AM), 1,5 carboxy-seminaphtorhodafluor acetoxymethyl ester (SNARF-1-AM), 3-(4,5-dimethylthiazol-2-yl)-2,5-diphenyltetrazolium bromide (MTT), triphenylphosphine oxide (TPPO, 99.4% purity), nicotine (99.8% purity), 9-phenanthrol (99.9% purity), l-arginine (99.0% purity), carbachol (99.7% purity), quinine (99.6% purity), 2-mercaptoethanol, 16% formaldehyde solution methanol-free, Hoechst-3334, Fluoromount-G^TM^, and Pluronic F-127 were obtained from Thermo Fisher Scientific. Fluorogenic Ca^2+^ sensitive dye Cal-520 AM was received from Biomol (Hamburg, Germany). Fetal bovine serum (FBS), penicillin–streptomycin, and trypsin/ethylenediaminetetraacetic acid were purchased from PAN-Biotech GmbH (Aidenbach, Germany). Homoeriodictyol (HED, purity >95%) was kindly provided by Symrise AG (Holzminden).^20^ Dimethyl sulfoxide (DMSO) was purchased from Carl Roth (Karlsruhe, Germany). PeqGOLD RNA Kit was obtained from VWR Peqlab (USA). All items for performing real-time qPCR (RT-qPCR) were purchased from BioRad (Feldkirchen, Germany). All reagents and siRNA for transient knockdown were obtained from Thermo Fisher Scientific (USA). Krebs-Ringer HEPES buffer (KRHB) is composed of 130 mM NaCl, 4.7 mM KCl, 1.3 mM CaCl_2_, 1.2 mM MgSO_4_, 1.2 mM KH_2_PO_4_, 11.7 mM d-glucose, and 10 mM HEPES; pH was adjusted to 7.4 with KOH. Na^+^ free KRHB is composed of 134.7 mM KCl, 1.3 mM CaCl_2_, 1.2 mM MgSO_4_, 1.2 mM KH_2_PO_4_, 11.7 mM d-glucose, and 10 mM HEPES; pH was adjusted to 7.4 with KOH. Caffeine (99.9% purity), histamine (99.6% purity), and concanavalin A biotin-conjugated were purchased from Sigma-Aldrich (Munich, Germany) and horse serum from Biowest (Nuaillé, France).

### Cell Culture

Human gastric tumor cells (HGT-1, RRID: CVCL_A609), provided by Dr. C. Laboisse, Nantes (France), were cultivated in DMEM containing 10% FBS and 1% penicillin and streptomycin at 37 °C, 5% CO_2_, and in a humidified atmosphere (standard conditions). Cells between passages 15 and 25 were used for all experiments.

### Cell Viability

To determine cell viability, 100,000 cells per well of a transparent 96-well plate were seeded the previous day. On the day of the experiment, the cells were washed with KRHB and then incubated for 60 min with the substances of interest. For the Na^+^ sensitive fluorescent dye SBFI (5 μM), the agonists caffeine (10 mM), l-arginine (50 mM), and histamine (1 mM), the antagonists HED (0.3 mM), TPPO (1 mM), nicotine (0.5 mM), and 9-phenanthrol (50 μM), and the solvent controls ethanol (1%) and DMSO (0.05%) were not found to affect cell viability (>88%). DMSO (100%) and quinine (5 mM) were used as negative controls (Figure S1). Cells were then washed again with KRHB and incubated with MTT (0.83 mg/mL in DMEM) for 15 min under standard conditions. Formazan formed was dissolved in DMSO, and the absorbance was measured (570 nm; reference 650 nm) on an Infinite M200 plate reader (Tecan, Switzerland). Untreated control cells (KRHB) were used to normalize and calculate the cell viability.

### Localization of TRPM4 and TRPM5 by Immunocytochemistry

For fluorescence labeling of HGT-1 cells, 50,000 cells were seeded at standard conditions for 24 h in a poly-d-lysine (10 μg/mL)-coated glass bottom 10-well plate (Greiner Bio-One GmbH, Leipzig, Germany). Then, the cells were incubated with PBS for 30 min before cell membrane staining with (1:2000) biotin-conjugated concanavalin A on ice for 1 h, followed by fixation with 4% formaldehyde for 10 min. After washing steps with PBS (3×, 5 min), 0.5% Triton-X-100 (1×, 5 min), and PBS (3×, 5 min), cells were incubated in a blocking solution with 5% horse serum and 0.3% Triton X-100 for 45 min to reduce the level of unspecific binding. Primary antibodies anti-TRPM4 antibody (RRID: AB_2040250) or anti-TRPM5 antibody (RRID: AB_2040252) (Alomone Labs, Jerusalem, Israel) (1:100) and corresponding blocking peptide (1:50) were incubated in blocking solution for approximately 1 h before applying to the cells. As a secondary antibody (Alexa Fluor 488 antirabbit IgG (RRID: AB_2536097) (Thermo Fisher Scientific Inc., USA) (1:1000) and for cell membrane staining streptavidin, Alexa Fluor 633 conjugate (RRID: AB_2313500) (Thermo Fisher Scientific Inc., USA) (1:1000) was selected and incubated for 1 h. The nucleus was labeled with Hoechst-33342 (1:2000) for 5 min, and the stained cells were embedded in Fluoromount-G^TM^. Immunofluorescence images were acquired and analyzed by confocal laser scanning microscopy using a Zeiss LSM 780 instrument (Carl Zeiss AG, Munich, Germany). For super-resolution imaging, an Airyscan detector was used.

### Determination of Intercellular Sodium Concentration Using Sodium-Sensitive Fluorescent Dye SBFI

To determine the intracellular sodium concentration, 50,000 cells per well of a black 96-well plate were seeded the previous day. On the experimental day, cells were washed with KRHB and then incubated with 5 μM Na^+^ sensitive fluorescent dye SBFI-AM (in KRHB containing 0.1% DMSO and 0.01% Pluronic F-127) for 2 h under standard conditions. Then, the cells were washed again with KRHB and incubated for another 30 min under standard conditions to allow complete de-esterification of the dye. After adding the test solutions, the plate was incubated in a FlexStation 3 (Molecular Devices, USA) for 60 min at 37 °C and fluorescence (excitation: 340/380 nm, emission: 500 nm) was recorded every 5 min. After calculation, according to the manufacturer’s protocol, fluorescence intensity was determined after 30 min and normalized to untreated control cells.

### Characterization of Intracellular Calcium Mobilization Using Calcium-Sensitive Fluorescent Dye Cal-520

To measure intracellular calcium mobilization, 50,000 cells per well of a black 96-well plate with a transparent bottom were seeded the previous day. On the experimental day, cells were washed with KRHB and then incubated with 1 μM Cal-520 AM (in KRHB containing 0.02% DMSO and 0.004% Pluronic F-127) for 2 h under standard conditions. Cells were then washed again with KRHB and incubated at 37 °C in a FlexStation 3. Fluorescence (excitation: 495 nm, emission: 515 nm) was recorded at 1 s intervals. After 60 s, the test substances were added in an automated setting and the fluorescence was recorded continuously. The fluorescence intensity was normalized to the first 60 s of the measurement before addition of each substance.

### Determination of the Intracellular Proton Concentration

To examine the effects of the investigated compounds on the proton secretion activity of HGT-1 cells, 100,000 cells per well of a black 96-well plate were seeded the previous day and incubated under standard conditions. After washing with KRHB, cells were incubated for 30 min with 3 μM of the pH-sensitive fluorescent dye SNARF-1-AM as previously described.^5,21^ After another washing step, the cells were stimulated with the test substances, and their intracellular proton secretion was determined using a FlexStation 3. The intracellular proton index (IPX) calculated from the measured data reflects the change in proton concentration compared to that of untreated control cells. Negative IPX values represent a stimulation of proton secretion, while positive IPX values indicate an inhibition of proton secretion.

### Determination of Gene Expression via RT-qPCR

For gene expression studies, 500,000 cells per well of a 24-well plate were seeded the previous day. On the day of the experiment, the cells were washed with PBS and incubated with the appropriate test solutions. This was followed by lysis of the cells and RNA isolation according to the manufacturer’s protocol using the peqGOLD RNA Kit (VWR Peqlab, USA). When the general expression of TRP channels was examined, the cells were directly lysed, and the RNA was extracted. The concentration of RNA was determined on NanoDrop One^c^ (Thermo Fisher Scientific Inc., USA). According to the manufacturer’s instructions, removing gDNA contaminants and synthesizing cDNA was performed using iScript gDNA Clear cDNA Synthesis Kit (BioRad, Feldkirchen, Germany). RT-qPCR was performed using SsoAdvanced Universal SYBR Green Supermix (Bio-Rad Laboratories, Inc., USA) and the previously published primers.^22^ All determined gene expressions were normalized to those of the reference genes GAPDH^23^ and PPIA^24^.

### Measurement of Intracellular Sodium Concentrations via LA–ICP–MS

To validate intracellular sodium concentrations in HGT-1 cells via single-cell laser ablation–inductively coupled plasma–mass spectrometry (LA–ICP–MS), 20,000 cells per well of a Cellview cell culture slide (Greiner Bio-One, Austria) were seeded the previous day. On the day of the experiment, the cells were washed with KRHB and then incubated with caffeine (10 mM) or l-arginine (50 mM) for 30 min under standard conditions. This was followed by another washing step with KRHB and drying of the slides for 2 h under standard conditions.

All laser ablation measurements were carried out with an Iridia 193 nm laser ablation system (Teledyne CETAC Technologies, USA), coupled to a NexION 5000 multiquadrupole ICP–MS instrument (PerkinElmer, USA). The laser ablation system was equipped with a cobalt long pulse ablation cell and connected to the ICP–MS via the Aerosol Rapid Introduction System (ARIS). Helium was used as a carrier gas (0.3 L/min) for aerosol transport from the ablation cell to the ICP. The LA–ICP–MS conditions were optimized on a daily basis using the NIST SRM 612 glass certified reference material (National Institute of Standards and Technology, USA). The nebulizer gas flow (∼0.92–0.98 L/min) was fine-tuned, generating maximum ^140^Ce^+^ signals, low oxide formation based on ^232^Th^+16^O^+^ (<100) and low elemental fractionation based on the ^238^U^+^/^232^Th^+^ ratio (∼1). The quadrupole ion deflector (QID) parameters were optimized by adjusting the QID lens scanning system for maximum efficiency, measuring ^7^Li^+^, ^24^Mg^+^, ^115^In^+^, ^140^Ce^+^, ^208^Pb^+^, and ^238^U^+^ as representatives of the entire mass spectrum. A radio frequency power of 1600 W, an auxiliary argon gas flow rate of 1.2 L/min, and a plasma gas flow rate of 16 L/min were used. The LA–ICP–MS imaging experiments were measured in the MS/MS standard mode (dwell time ^23^Na^+^: 50 ms) and ablating an area of ∼200 × 175 μm line by line (unidirectional, laser off between rows). Quantitative ablation was achieved by selecting a fluence of 1.0 J/cm^2^ with a fixed dosage of 9, at a repetition rate of 162 Hz and using a 3 μm circle spot size. The integration and readout rate were optimized to match the laser ablation repetition rate. The ICP–MS signal, received from Syngistix v.3.3 (PerkinElmer, USA) was synchronized with the timestamps in the laser log files from Chromium v.3.1 (Teledyne CETAC Technologies, USA) and further processed with the laser ablation data software HDIP-v1.7.1 (Teledyne CETAC Technologies, USA). Single cells were extracted as regions of interest and visualized via HDIP-v1.7.1. The complete instrument settings are listed in Table S1.

### Transient Knockdown of TRPM4 and TRPM5 in HGT-1 Cells

To quantitate the functional role of the two ion channels TRPM4 and TRPM5, a knockdown (kd) was performed using siRNA. The procedure was analogous to the already published protocol.^25^ Stealth siRNA TRPM4 (HSS123260) and TRPM5 (HSS179077) were purchased from Thermo Fisher Scientific (USA).

### Stable TAS2R43 Knockout Using CRISPR-Cas9

For experiments with TAS2R43 homozygote KO HGT-1 cells, the same clone was used as previously published.^5^

### Statistical Analysis

Unless otherwise described, all data are presented as box plots with 10th and 90th percentiles. Data points outside of this range are indicated as dots. At least four independent biological replicates were used for each experiment. Data were subjected to a Nalimov outlier test and then analyzed via a one-way ANOVA Holm-Šidák *post hoc* test. Different *p* values are indicated with asterisks according to the following scheme: *p ≤ 0.05, **p ≤ 0.01, ***p ≤ 0.001, and ****p ≤ 0.0001.

## Results

### TRPM4 and TRPM5 are Expressed in HGT-1 Cells

**Figure 1:**
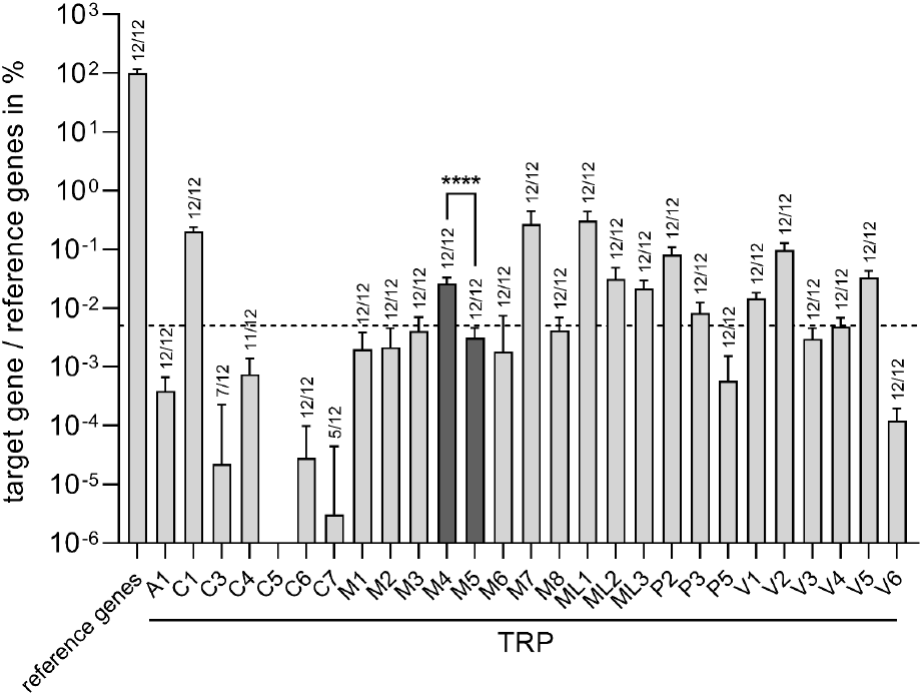
Representation of gene expression of TRP channels in HGT-1 cells normalized to the reference genes GAPDH and PPIA. Four different passages in three technical replicates were examined. Numbers above the bars indicate in how many samples the specific PCR product was found. The dashed line represents the mean gene expression of all 27 TRP channels. Data are shown as mean ± SEM, *n* = 4, t. r. = 3.

First, the RNA expression of all 27 members of the human TRP superfamily was investigated via RT-qPCR. Except for TRPC5, the RNA expression of all TRP channels could be detected in HGT-1 cells (Figure 1). Regarding the respective transcript levels, TRPM4 revealed a slightly, yet statistically significant, higher RNA expression level compared with TRPM5 (*p* ≤ 0.0001; Figure 1).

**Figure 2:**
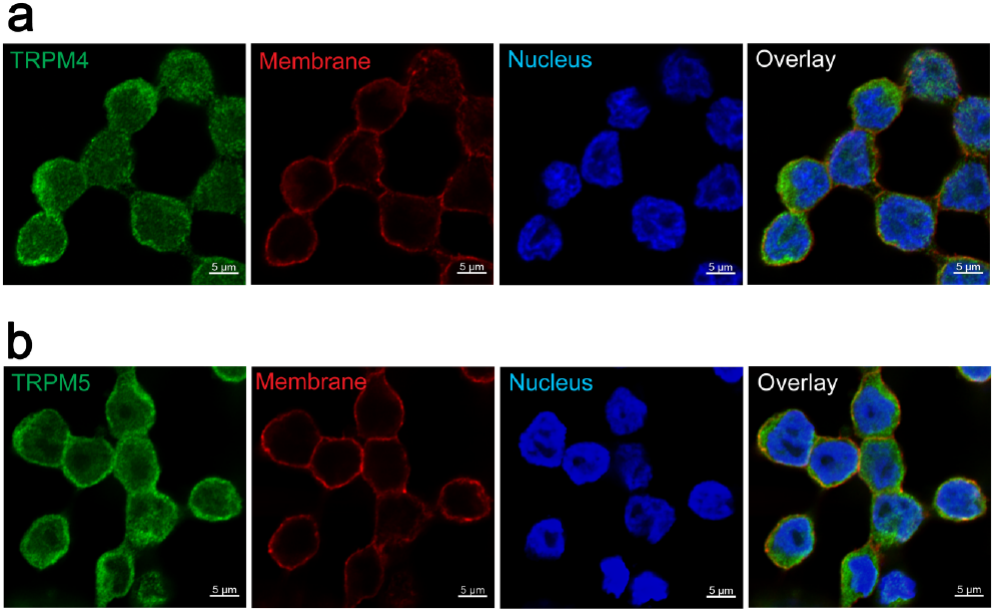
TRPM4 and TRPM5 are expressed in HGT-1 cells. Super-resolution images were acquired with the Airyscan detector of Zeiss LSM 780. Ion channel expression is detected by anti-TRPM4 antibody (a) and anti-TRPM5 antibody (b) in combination with an Alexa Fluor 488 antirabbit IgG (green). The nucleus is visualized by Hoechst-33342 (blue), and the plasma membrane by biotin-conjugated concanavalin A in combination with Alexa Fluor 633 conjugate (red). Scale: 5 μm.

Following our hypothesis, we next analyzed whether TRPM4 and TRPM5 are expressed in HGT-1 cells on the protein level via immunocytochemistry. Staining of HGT-1 cells for TRPM4 and TRPM5 led to a fluorescence signal in the plasma membrane and the cytoplasm (Figure 2a,b). Application of the respective blocking peptides diminished these signals (Figure S2), thereby confirming the expression of TRPM4 and TRPM5 on the protein level in HGT-1 cells.

### Bitter Compounds but Not Histamine Increased Intracellular Fluorescence Signals of the Na^+^ Sensitive Dye SBFI

**Figure 3:**
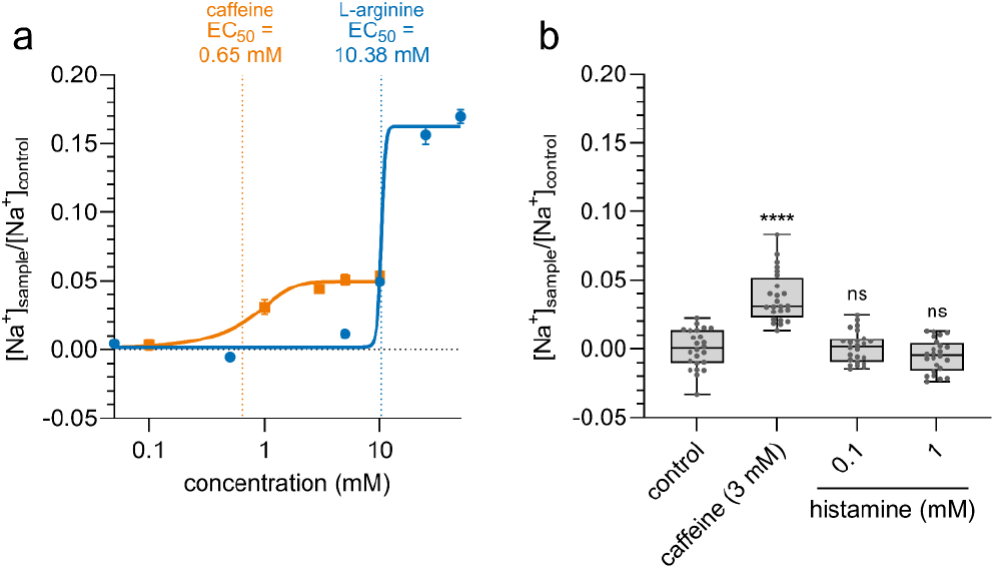
(a) Caffeine and l-arginine lead to a concentration-dependent Na^+^ influx into HGT-1 cells. For caffeine, the EC_50_ is reached at 0.65 mM, whereas the EC_50_ of l-arginine is 10.38 mM. (b) Treatment of cells with caffeine (3 mM) increases intracellular Na^+^ concentrations compared to untreated control cells. However, the Na^+^ concentration of histamine-treated cells (0.1 or 1 mM) does not differ from untreated cells. Statistics: *n* = 4, t. r. = 6, one-way ANOVA Holm-Šidák *post hoc* test; significant differences are expressed with ****p ≤ 0.0001.

According to our hypothesis, a bitter signaling pathway like in taste cells should lead to Ca^2+^ mobilization from the endoplasmic reticulum and, consequently, to TRPM4/M5-mediated Na^+^ influx after applying bitter compounds via activation of TAS2Rs expressed in HGT-1 cells. Therefore, we assessed whether incubation of HGT-1 cells with different concentrations of the TAS2R agonists caffeine and l-arginine for 30 min results in an increase in the intracellular Na^+^-dependent fluorescence of the dye SBFI. The two compounds were selected based on their different TAS2R target profile and their ability to induce proton secretion in HGT-1 cells. While caffeine is the prototypical coffee bitter substance activating 5 of the 25 human TAS2Rs (TAS2R7, 10, 14, 43, and 46)^26^ and demonstrated to induce gastric acid secretion in healthy subjects,^5^l-arginine is the third most bitter amino acid^27^ and also a potent inducer of proton secretion in HGT-1 cells.^28^l-arginine induced a robust increase of the cytosolic Ca^2+^ concentration (Figure S3), while caffeine application resulted in a notable dip in the fluorescence intensity (Figure S3), probably due to its well-known quenching properties.^29^ However, concentration–response analyses revealed a caffeine-concentration-dependent increase in SBFI-fluorescence intensity. For the caffeine-concentration-dependent rise in the intracellular Na^+^ concentration, an EC_50_ value of 0.65 mM was calculated (Figure 3a orange line). The bitter compound l-arginine also induced a concentration-dependent increase in the intracellular SBFI-fluorescence (Figure 3a blue line) with an EC_50_ value of 10.38 mM. However, the efficacy was higher for l-arginine compared to that of caffeine (*p* ≤ 0.0001). By incubating cells from four different cell passages with varying concentrations of caffeine (1, 3, 5, and 10 mM), we could show that these changes in the SBFI-fluorescence intensity are stable and repeatable (Figure S4). Since histamine stimulates gastric acid secretion in HGT-1 cells TAS2R-independent^30^ via the H_2_ receptor, HGT-1 cells were incubated with two different histamine concentrations, and the SBFI-fluorescence was analyzed. As hypothesized, stimulating the cells with histamine did not increase the SBFI-fluorescence signal (*p* > 0.60; Figure 3b), indicating that the increased SBFI-fluorescence is TAS2R dependent.

### Pharmacological Inhibition of TAS2Rs and Genetic Knockout of TAS2R43 Diminishes the Na^+^ Influx

**Figure 4:**
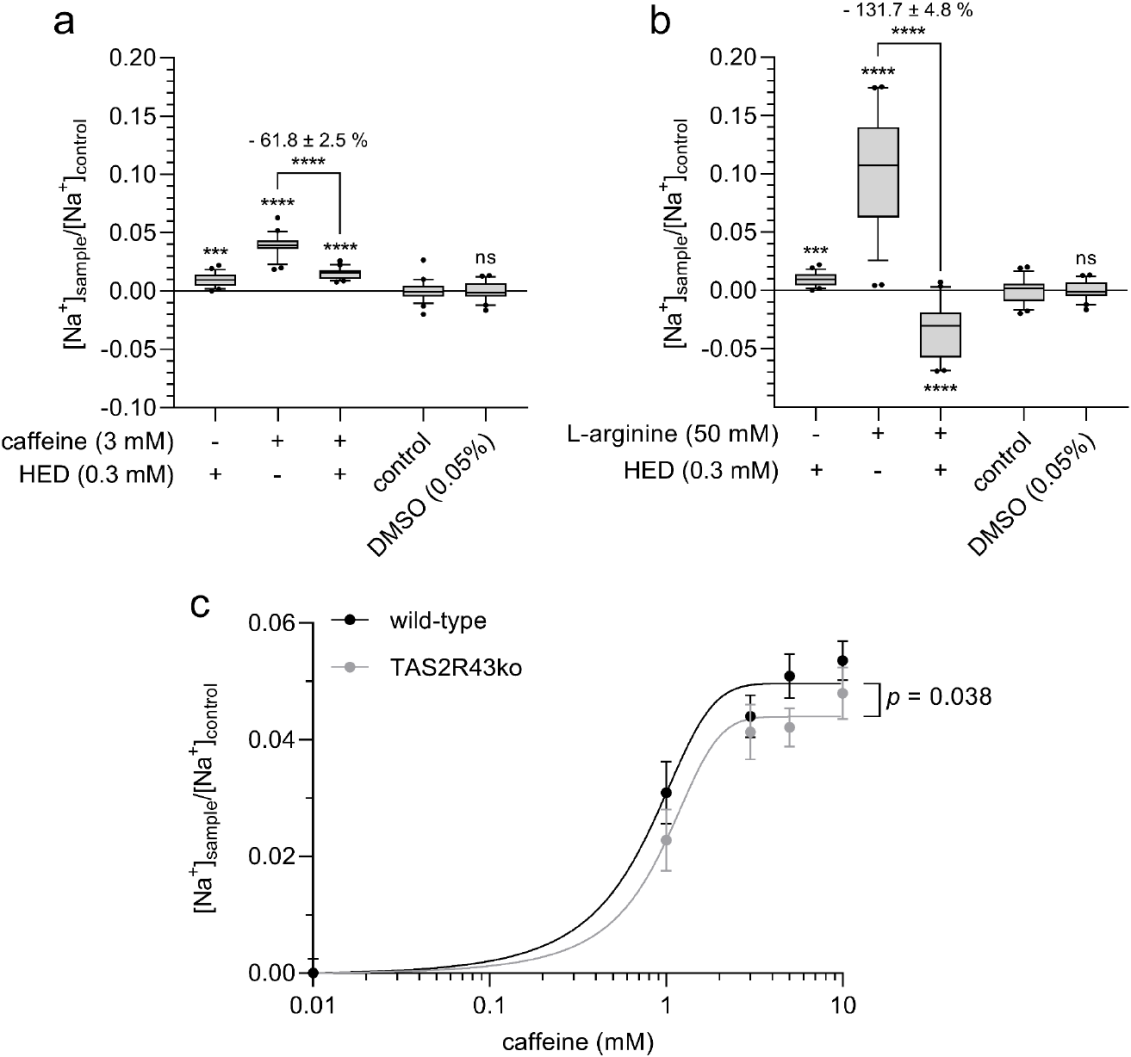
Pharmacological inhibition of TAS2Rs by homoeriodictyol (0.3 mM) leads to a reduction in (a) caffeine- (3 mM) and (b) l-arginine-induced (50 mM) Na^+^ influx in cells. (c) Stable TAS2R43 knockout by CRISPR-Cas9 reduces caffeine-induced Na^+^ influx into cells at different caffeine concentrations (0.01–10 mM). Statistics: *n* = 4, t. r. = 6, one-way ANOVA Holm-Šidák *post hoc* test; significant differences are expressed with ***p ≤ 0.001, ****p ≤ 0.0001.

We next aimed to analyze whether the influx of Na^+^ is initiated via binding of bitter compounds to TAS2Rs provoking taste-cell-like signaling. For this, the bitter-masking TAS2R antagonist homoeriodictyol (HED), which has been described to reduce the bitter taste of caffeine in human sensory panels by up to 49%,^20,31^ as well as the caffeine-evoked proton secretion in HGT-1 cells^5^ by inhibiting TAS2R20/31/43/50,^5^ was applied. When HGT-1 cells were coincubated with caffeine and HED, the caffeine-induced Na^+^ influx (+3.89 ± 0.20%; *p* ≤ 0.0001) was reduced by 61.8 ± 2.5% (*p* ≤ 0.0001; Figure 4a). Incubation of the cells with 0.3 mM HED alone led to a small, yet statistically significant, influx of Na^+^ (+0.96 ± 0.12%; *p* ≤ 0.0001). The respective solvent control (0.05% DMSO) had no impact (*p* > 0.99). In addition to caffeine, bitter-tasting amino acid l-arginine also increased Na^+^ influx into HGT-1 cells (+10.24 ± 1.00%; *p* ≤ 0.0001; Figure 4b). This stimulatory effect was reversed by coincubation with HED (−131.7 ± 4.8%; *p* ≤ 0.0001) so that coincubated cells revealed a lower intracellular Na^+^ concentration than untreated cells.

Since HED targets TAS2R43, and caffeine has been shown to activate, among others, TAS2R43, we next analyzed whether the Na^+^ influx upon caffeine stimulation of HGT-1 cells is reduced in CRISPR/Cas TAS2R43 knockout (TAS2R43ko) cells, which were established previously.^5^ Upon stimulation with different concentrations of caffeine, the TAS2R43ko cells revealed a lower Na^+^ influx compared to that of the wild-type cells (*p* = 0.038; effect size −17.30 ± 6.17% for 5 mM caffeine) (Figure 4c).

### The Increased Intracellular SBFI-Fluorescence is Na^+^ Specific and Results from Na^+^ Influx

**Figure 5:**
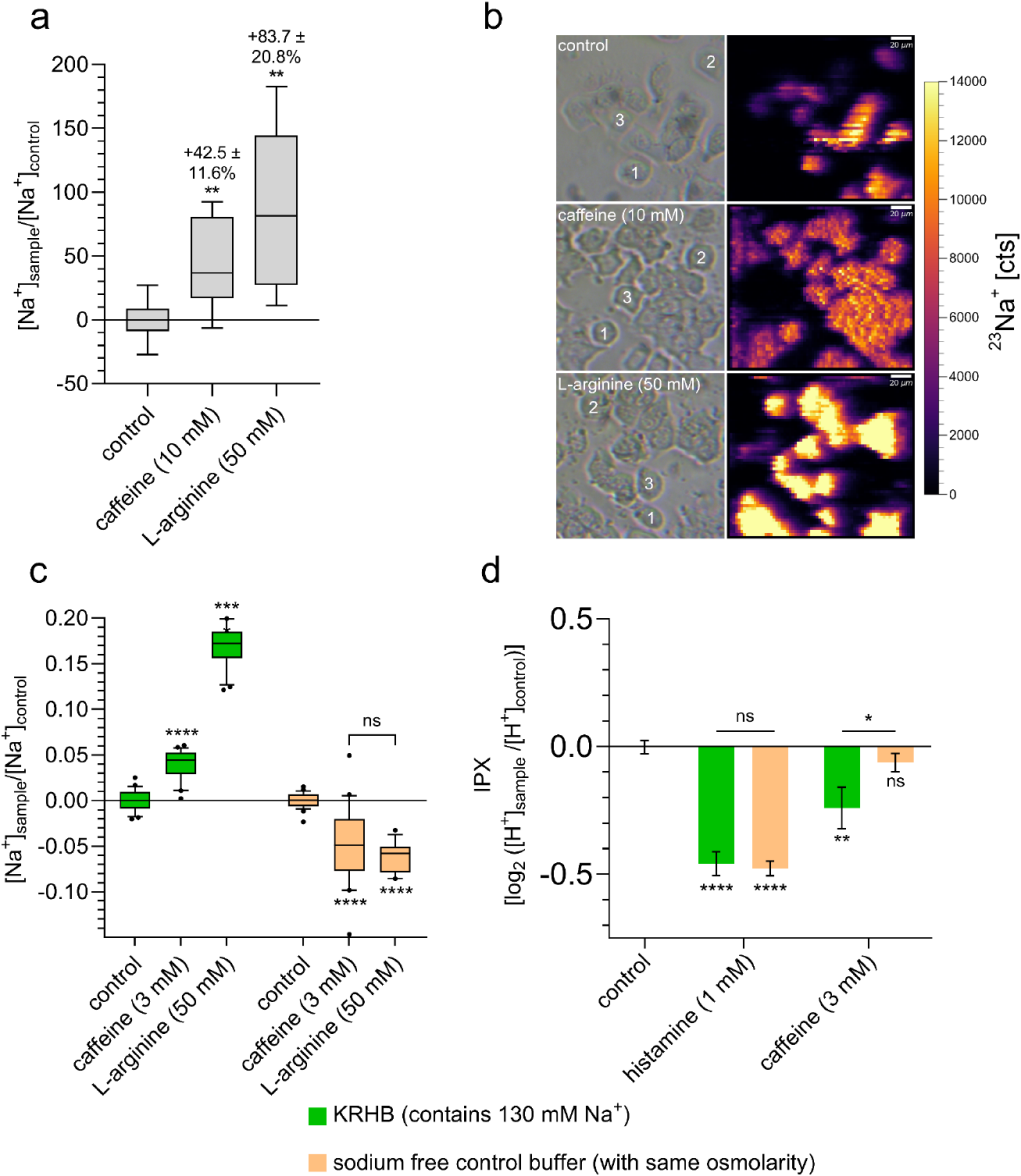
(a) Measurement of intracellular Na^+^ concentration by LA–ICP–MS validated the results obtained by using Na^+^ sensitive fluorescent dye. Incubation of HGT-1 cells with caffeine (10 mM) and l-arginine (50 mM) increases Na^+^concentration in the cell. (b) Bright-field images of HGT-1 cells (left). Signal intensity maps of ^23^Na^+^ in HGT-1 cells obtained by LA–ICP–MS imaging (right). LA-Parameters: fluence: 1.0 J/cm^2^; repetition rate: 162 Hz; spot size: 3 μm circle; fixed dosage mode: 9; dwell time ^23^Na^+^: 50 ms. (c) Treatment of cells with caffeine (3 mM) or l-arginine (50 mM) in Na^+^-containing buffer (KRHB) resulted in an increase in the Na^+^-dependent fluorescence signal within the cells. Incubation with the same substances but in Na^+^-free buffer did not increase the Na^+^ concentration within the cells. (d) Similar results were obtained when measuring caffeine-induced stimulation of proton secretion. Here, treatment of cells with caffeine in the presence of Na^+^ in the external medium resulted in increased proton secretion, whereas this was no longer detectable when measured in Na^+^-free buffer. However, treatment of the cells with histamine (1 mM) resulted in stimulation of proton secretion in both cases. In (d), data are shown as mean ± SEM. Statistics: *n* = 4–5, t. r. = 6, one-way ANOVA Holm-Šidák *post hoc* test; significant differences are expressed with *p ≤ 0.05, **p ≤ 0.01, ****p ≤ 0.0001.

Next, we wanted to confirm that SBFI-fluorescence is a suitable indicator for intracellular Na^+^ concentrations in HGT-1 cells. Therefore, we determined intracellular Na+ concentrations via single-cell LA–ICP–MS after incubating HGT-1 cells with concentrations for which the most pronounced effects of caffeine and l-arginine are demonstrated in Figure 3a for the respective solvent control. These analyses revealed that incubation of HGT-1 cells with 10 mM caffeine (+42.5 ± 11.6%; *p* ≤ 0.01) or 50 mM l-arginine (+83.7 ± 20.8%; *p* ≤ 0.01) leads to an increase in intracellular Na^+^ concentrations (Figure 5ab), validating the measurements using the Na^+^ sensitive fluorescent dye SBFI.

To verify that the increased intracellular Na^+^ concentrations are based on Na^+^ influx from the extracellular space, HGT-1 cells were stimulated with either 3 mM caffeine or 50 mM l-arginine in KRHB containing 130 mM Na^+^ and in nominal Na^+^-free KRHB, respectively, followed by monitoring the resulting SBFI-fluorescence. The presence of Na^+^ in the KRHB solution led to the already observed increase of the intracellular Na^+^ concentration after incubation with 3 mM caffeine (+4.00 ± 0.32%; *p* ≤ 0.0001) and 50 mM l-arginine (+16.97 ± 0.47%; *p* ≤ 0.0001; Figure 5c, left), whereas incubation of HGT-1 cells with caffeine and l-arginine in a nominal Na^+^-free KRHB even led to lower intracellular Na^+^ concentrations compared to untreated control cells (−4.73 ± 0.85% and −6.22 ± 0.46%, respectively; *p* ≤ 0.0001; Figure 5c, right), confirming that incubation of HGT-1 cells with the bitter compounds caffeine and l-arginine induces an influx of Na^+^ from the extracellular space. Also, the proton secretion induced by caffeine was reduced in nominal Na^+^-free buffer (*p* ≤ 0.05), indicating the involvement of Na^+^ influx in this mechanism (Figure 5d). This was not the case when proton secretion was TAS2R-independently induced by histamine.

### The Na^+^ Influx Induced by Bitter Compounds is TRPM4 and TRPM5 Dependent

**Figure 6:**
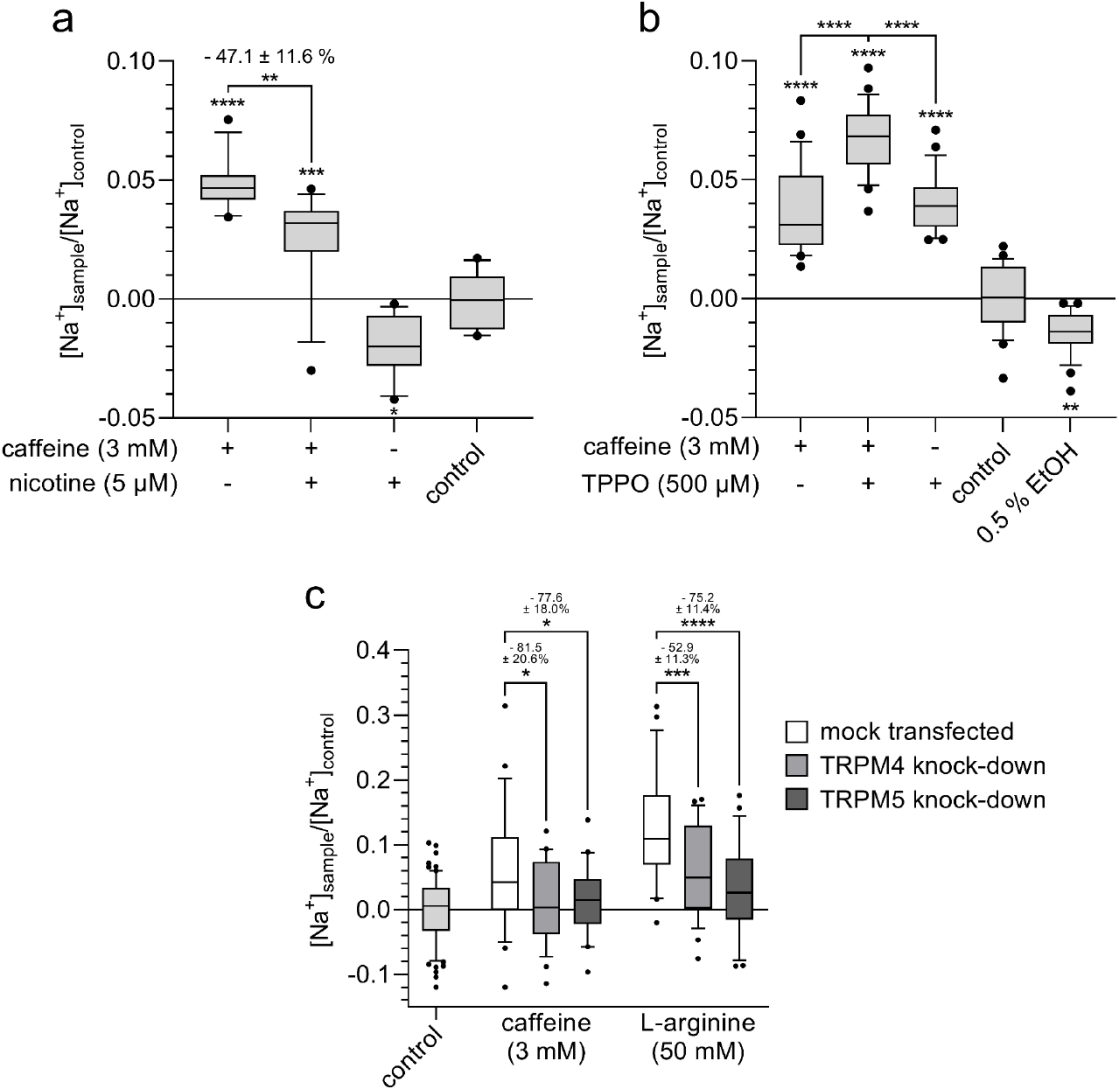
Whereas coincubation of cells with caffeine (3 mM) and the (a) TRPM5 inhibitor nicotine (5 μM) leads to a reduction in Na^+^ influx, (b) simultaneous treatment with the TRPM5 inhibitor TPPO (500 μM) increases Na^+^ influx. (c) Transient reduction of TRPM4 or TRPM5 expression by siRNA leads to decreased Na^+^ influx upon stimulation with caffeine (3 mM) or l-arginine (50 mM), respectively, compared with mock-transfected cells. Statistics: *n* = 4, t. r. = 6, one-way ANOVA Holm-Šidák *post hoc* test; significant differences are expressed with *p ≤ 0.05, **p ≤ 0.01, ***p ≤ 0.001, ****p ≤ 0.0001.

To test whether the Na^+^ influx elicited by the binding of bitter compounds to TAS2Rs is mediated via TRPM4 and/or TRPM5, different antagonists of TRPM4 and TRPM5 were applied (Figure 6). Pharmacological inhibition of TRPM5 with nicotine^32^ led to a lower Na^+^ influx in HGT-1 cells when costimulated with 3 mM caffeine (−47.1 ± 11.6%; *p* ≤ 0.01; Figure 6a). Notably, this effect could not be detected, when the TRPM5-specific inhibitor TPPO^33^ was applied (Figure 6b). Since the TRPM4-specific inhibitor 9-phenanthrol^34^ could not be entirely removed from the cells and reveals spectral overlapping with the Na^+^ dye SBFI, pharmacological inhibition of TRPM4 was, for technical reasons, unsuccessful (data not shown).

Since the pharmacological inhibition experiments provided inconclusive results, TRPM4 and TRPM5 siRNA knockdown (kd) experiments were performed. The mean knockdown efficiency was determined as −42.1 ± 7.0% (*p* ≤ 0.01) for TRPM4 and 68.7 ± 4.9% (*p* ≤ 0.0001) for TRPM5 (Figure S5). Knockdown of TRPM4 or TRPM5 independently reduced the Na^+^ influx induced by caffeine (−81.5 ± 20.6% for TRPM4kd cells; *p* ≤ 0.05 and −77.6 ± 18.0% for TRPM5kd cells; *p* ≤ 0.05) and l-arginine (−52.9 ± 11.3% for TRPM4kd cells; *p* ≤ 0.001 and −75.2 ± 11.4% for TRPM5kd cells; *p* ≤ 0.0001), thereby confirming the involvement of TRPM4 and TRPM5 in the Na^+^ influx in HGT-1 cells upon stimulation with bitter compounds (Figure 6c). No difference was observed regarding the effect size for TRPM4 vs TRPM5 (*p* = 0.90 for caffeine, *p* = 0.15 for l-arginine), indicating that both channels contribute about equally to the Na^+^ influx.

Also, bitter compounds impact not only TRPM4 and TRPM5 on the functional level but also on the transcript level. Incubation of the cells with 3 mM caffeine led to reduced transcript levels of TRPM4 after only 10 min, while TRPM5 transcript levels were reduced after 30 min (Figure S6).

### Activation of Muscarinic Acetylcholine Receptor Leads to Na^+^ Influx by TRPM4 and TRPM5

**Figure 7:**
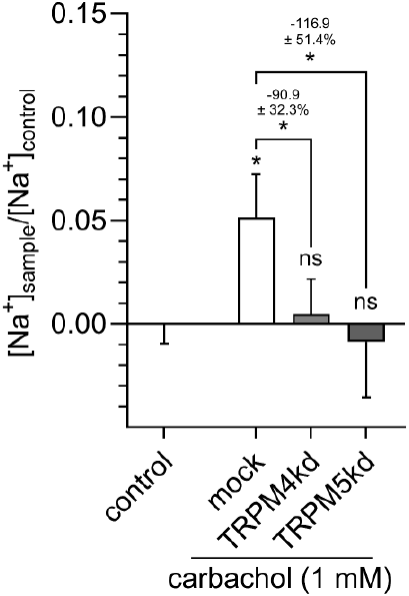
Treating mock-transfected HGT-1 cells with the muscarinic acetylcholine receptor agonist carbachol (1 mM) increases intracellular Na^+^ concentrations. This increase cannot be detected in TRPM4 or TRPM5 knockdown cells. Data are shown as mean ± SEM, *n* = 4, t. r. = 6, statistics: one-way ANOVA Holm-Šidák *post hoc* test; significant differences are expressed with *p ≤ 0.05.

Since activation of the muscarinic acetylcholine receptor (M_3_) leads to a mobilization of Ca^2+^ from the endoplasmic reticulum to the cytosol, and both TRPM4 and M5 are activated by increased cytosolic Ca^2+^ concentrations, we next investigated whether activation of the cholinergic pathway also triggers TRPM4- and TRPM5-mediated Na^+^ influx into HGT-1 cells. Therefore, the cells were treated with carbachol, a well-known structural analog of acetylcholine.^35^ As hypothesized, exposure of the cells to 1 mM carbachol led to Ca^2+^ mobilization (Figure S3) and increased the intracellular Na^+^ concentration by +5.14 ± 2.05% (*p* ≤ 0.05). Again, the involvement of TRPM4 and M5 was investigated by knockdown experiments. A reduction in intracellular Na^+^ concentration of −90.9 ± 32.3% (*p* ≤ 0.05) for TRPM4kd and −116.9 ± 51.4% (*p* ≤ 0.05) for TRPM5kd cells, respectively, was detected (Figure 7).

### TRPM4- and TRPM5-Dependent Na^+^ Influx is Involved in Proton Secretion

**Figure 8:**
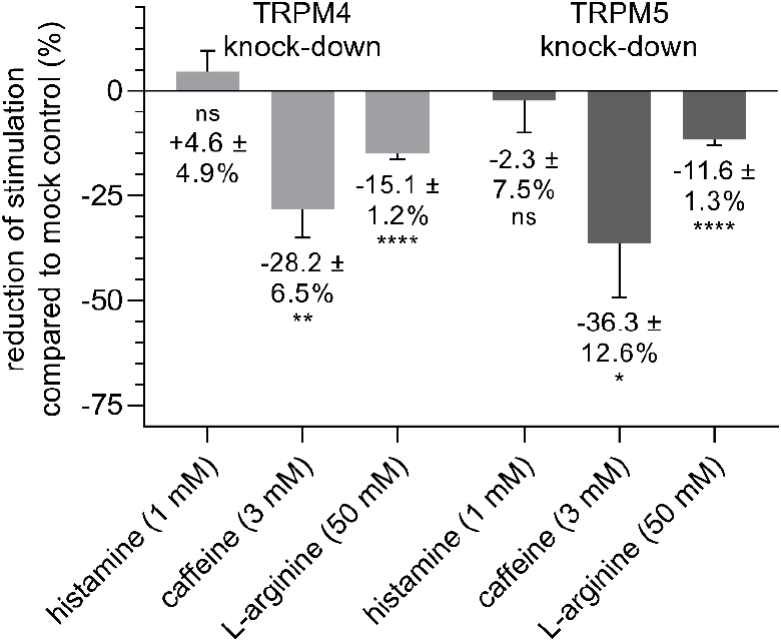
Transient knockdown of TRPM4 or TRPM5 leads to a reduction in the stimulation of proton secretion induced by caffeine (3 mM) or l-arginine (50 mM). Treatment with histamine (1 mM) did not result in differentiation between TRPM4 or TRPM5 knockdown and mock-transfected cells. Data are shown as mean ± SEM, *n* = 4, t. r. = 6, statistics: one-way ANOVA Holm-Šidák *post hoc* test; significant differences are expressed with *p ≤ 0.05, **p ≤ 0.01, ****p ≤ 0.0001.

Next, we hypothesized that the TRPM4/5-evoked Na^+^ influx induced by food constituents modulates the proton secretion of HGT-1 cells. For this purpose, the proton-secreting activity was analyzed after stimulation of wild-type cells as well as TRPM4kd and TRPM5kd cells with histamine, caffeine, and l-arginine, respectively (Figure 8). We could show that both TRPM4 or TRPM5 knockdown reduced the proton secretion upon stimulation with 3 mM caffeine (−28.2 ± 6.5%; *p* ≤ 0.01 and −36.3 ± 12.6%; *p* ≤ 0.05) and 50 mM l-arginine (−15.1 ± 1.2%; *p* ≤ 0.0001 and −11.6 ± 1.3%; *p* ≤ 0.0001), respectively. This result was confirmed for caffeine by applying TRPM5- (Figure S7a,b) and TRPM4-specific (Figure S7c) antagonists. In contrast, incubation of TRPM4kd or TRPM5kd cells with 1 mM histamine did not show any deviation from mock-transfected cells regarding proton secretion.

## Discussion

Gastric acid secretion is a vital, yet tightly regulated process. The physiological stimuli of proton secretion as key mechanisms of gastric acid formation include acetylcholine, gastrin, and especially, histamine. Apart from endogenous signals, food-derived bitter compounds have also been shown to induce proton secretion via binding to TAS2Rs located on parietal cells.^5^ However, the particular underlying signaling cascade, finally leading to proton secretion, has yet to be fully elucidated. In contrast, the mode of action of bitter compounds in taste cells located on the tongue has been very well described and includes activation of the TRP channels M4 and M5 leading to an influx of Na^+^.^8,10^ Here, we hypothesized a similar cellular signaling pathway of bitter compounds in the parietal cell line HGT-1, a well-established surrogate model of human parietal cells.^5,18^

First of all, we analyzed the expression of all members of the TRP superfamily on the RNA level and the expression of TRPM4 and M5 also on the protein level. We could demonstrate the expression of a variety of TRP-specific transcripts and, most importantly, the expression of TRPM4 and M5 on the protein level. Both channels are located in the cytoplasm and the plasma membrane, which may indicate regulation of cell surface expression via internalization in HGT-1 cells, which was shown before for the TRPM4 channel in a variety of cells.^36−38^ In the colorectal cancer cell line HCT116, increasing concentrations of intracellular Ca^2+^ led to the delivery of TRPM4-containing vesicles to the plasma membrane.^39^ This presumed regulation of TRPM4 and TRPM5 in HGT-1 cells could also explain the downregulation of their specific transcripts due to a stimulation of the cells with 3 mM caffeine for 10 and 30 min, respectively.

According to our hypothesis, TRPM4 and TRPM5 are functionally linked to TAS2Rs in HGT-1 cells. Therefore, we tested whether stimulation of the cells with bitter compounds leads to an influx of Na^+^. For this, the Na^+^ sensitive fluorescence dye SBFI was applied. Incubation of the cells with the food-derived bitter compounds caffeine, targeting the bitter receptors TAS2R7, 10, 14, 43, and 46,^26^ as well as l-arginine, targeting at least TAS2R1,^28^ led to concentration-dependent increases in SBFI-fluorescence. Caffeine showed a higher potency with an EC_50_ value of 0.65 mM but a lower efficacy (maximal efficacy: 5.0%) than l-arginine (EC_50_: 10.38 mM; maximal efficacy: 16.3%). The higher potency of caffeine for inducing a Na^+^ influx compared to l-arginine can be linked to the lower bitter recognition threshold of caffeine, which has been determined as ranging from 0.7 to 3.7 mM^40,41^ compared to 75 mM for l-arginine.^42^ To our knowledge, no data are available on whether the bitter perceptions of l-arginine and caffeine in equimolar concentrations differ.

By additionally applying the fluorescence-independent and direct method single-cell LA–ICP–MS, we could clearly demonstrate that the SBFI-fluorescence not only is a suitable indicator for intracellular Na^+^ concentrations in HGT-1 cells but furthermore can be used in a semiquantitative manner. In addition, the observed increased intracellular Na^+^ concentrations result from an influx of Na^+^ since the SBFI-fluorescence did not increase when HGT-1 cells were stimulated with caffeine and l-arginine in a nominal Na^+^ free buffer. These results confirm former analyzes using SBFI to measure Na^+^ fluxes in parietal cells from New Zealand White rabbits, although the ion channel responsible was not identified.^43^ Also, the detected Na^+^ influx is linked to TAS2Rs since stimulating HGT-1 cells with histamine, whose signaling pathway in parietal cells is mediated via the H_2_ receptor, did not impact intracellular Na^+^ concentrations. In fact, coincubating HGT-1 cells with caffeine or l-arginine and the bitter-masking compound HED led to lower (−61.8 ± 2.5% for caffeine and −131.7 ± 4.8% for l-arginine) intracellular Na^+^ concentrations. These findings strongly suggest that Na^+^ influx in HGT-1 cells upon stimulation with bitter compounds is mediated via the interaction with TAS2Rs. Interestingly, stimulation of the cells with HED alone did induce a Na^+^ influx (+0.96 ± 0.12%; *p* ≤ 0.0001), which might be due to the fact that HED was identified not only as an antagonist for the TAS2Rs 31, 43, 20, and 50 but also as an agonist for TAS2R14 and TAS2R39.^44^ According to this knowledge, the activation of TAS2R14 and/or TAS2R39 by HED would lead to an influx of Na^+^, as measured.

As TAS2R43 can be activated by caffeine,^26^ the effect of caffeine on Na^+^ influx was further investigated using TAS2R43ko HGT-1 cells.^5^ The results obtained further evidenced the contribution of TAS2Rs in initiating the Na^+^ influx since the TAS2R43 knockout cells demonstrated a decreased Na^+^ influx as compared to the HGT-1 wild-type cells (Figure 4c). The remaining Na^+^ influx in TAS2R43 knockout cells is very likely caused by the activation of other TAS2Rs (7, 10, 14, and 46), which are also targeted by caffeine.^26^ Since we hypothesized a non TAS2R43-specific effect here, we also tested l-arginine, which is not a ligand for this receptor. The results presented in Figure 4c support this hypothesis. Which of the TAS2Rs has the greatest impact in stimulation Na^+^ influx has to be investigated in future studies. However, these results implicate that, as in taste cells, the Na^+^ influx is activated downstream of TAS2Rs.

Pharmacological inhibitors were applied to verify the involvement of TRP channels M4 and M5 in the Na^+^ influx triggered by bitter compounds. Co-incubation of the cells with caffeine and the TRPM5 inhibitor nicotine led to a nearly 50% lower Na^+^ influx than cells treated with caffeine. Incubation of the cells with nicotine alone resulted in lower intracellular Na^+^ concentrations compared with control cells. This may indicate a more general function of TRPM5 in the control of intracellular Na^+^ concentrations. In addition, nicotine may interact with other receptors in HGT-1 cells, leading to the observed effects. However, contrarily, application of the TRPM5-specific inhibitor TPPO^33^ alone led to a Na^+^ influx in HGT-1 cells comparable to caffeine. In contrast, coapplication with TPPO and caffeine doubled the impact of the single compounds. Here, we hypothesize TPPO to interact nonselectively with other ion channels in HGT-1 cells, which has already been shown for Chinese ovarian hamster (CHO) cells expressing P2X2/P2X3 receptors.^45^

Nevertheless, genetic knockdown of TRPM4 and TRPM5 decreased the Na^+^ influx in HGT-1 cells upon stimulation with caffeine and l-arginine by 52.9–81.5%. These results show that the Na^+^ influx in HGT-1 cells upon stimulation with food-derived bitter compounds is mediated via TRPM4 and TRPM5. A significant difference between the impact of TRPM4 and TRPM5 was not detected. Thus, both channels account for the Na^+^ influx about equally. This also reflects the situation in taste cells, where TRPM4 and TRPM5 act downstream of TAS2Rs and contribute to a similar degree to the Na^+^ influx upon stimulation with bitter compounds.^8^ However, we cannot fully rule out the possibility that other Na^+^ permeable ion channels, like the epithelial sodium channel (ENaC), may also contribute, albeit to a smaller extent, to the Na^+^ influx.^46^ On the other hand, activation of ENaC by bitter compounds has not been described so far. Nonetheless, TRPM4 and TRPM5 are also involved in the Na^+^ influx induced by the structural acetylcholine analogue carbachol since the knockdown of either ion channel decreased the carbachol-induced proton secretion of HGT-1 cells up to 100%. This finding aligns with former results by Negulescu et al., who detected a Na^+^ influx in parietal cells from New Zealand White rabbits upon stimulation with 100 μM carbachol.^43^ One open question to be elucidated in future studies is whether carbachol targets, in addition to the M_3_ receptor, e.g., taste receptors.

In nonsensory tissue other than parietal cells, bitter substances stimulated CCK secretion from enteroendocrine STC-1 cells with the participation of TRPM5.^47^ Shah et al. observed that bitter compounds elicited a TRPM5-specific inward current, which might be responsible for the release of CCK.^48^ In *trpm5*^–/–^ mice, not only the glucose-induced insulin secretion was reduced, but also the l-arginine-induced insulin secretion.^49^ This finding was confirmed for l-arginine-induced insulin secretion from rat β-cells using the TRPM5-specific inhibitor TPPO.^50^ TRPM4, on the other hand, was shown to be involved in glucose-induced insulin secretion but not l-arginine-induced insulin secretion.^51^ However, l-arginine-induced insulin secretion is mediated, at least partly, via GPRC6A,^52^ and participation of bitter taste receptors in this process has, to our knowledge, not been demonstrated so far. Based on our findings, TRPM4 and M5 are cellular targets for taste-active food ingredients, although knowledge regarding food constituents that are capable of directly activating or inhibiting TRPM4 or TRPM5 is scarce. For example, TRPM5 was shown to be modulated by the sweetener stevioside,^53^ which could imply an impact of this taste-active compound on gastric acid secretion. Furthermore, the results presented may raise the question of whether the observed physiological functions of taste-active food constituents, especially those for which beneficial health effects have been shown, e.g., coumaric acid^54^ and polyphenols,^55−57^ are mediated via a taste-like signaling pathway in nonsensory tissue.

**Figure 9:**
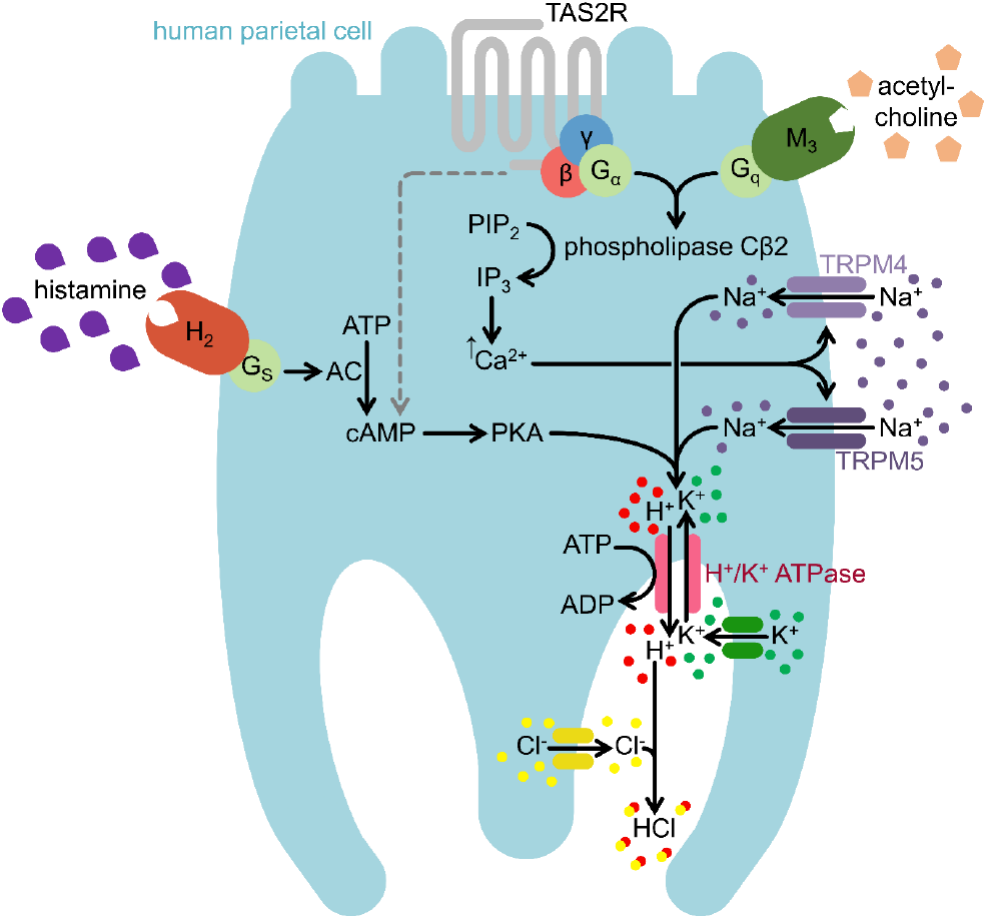
TRPM4 and TRPM5 mediate Na^+^ influx upon stimulation with bitter compounds and carbachol (acetylcholine) in parietal cells from the cell line HGT-1 as major signaling pathway. AC: adenylylcyclase; ADP: adenosinediphosphate; ATP: adenosintriphosphate; cAMP: cyclic adenosinmonophosphate; IP_3_: inositol-1,4,5-trisphosphate; PIP_2_: phosphatidylinositol-4,5-bisphosphate; PKA: protein kinase A; H_2_: histamine-H_2_-receptor; M_3_: muscarinic acetylcholine receptor; TAS2R: family 2 of taste receptors; TRPM4: transient receptor potential M4; TRPM5: transient receptor potential M5; H^+^/K^+^ ATPase: hydrogen potassium ATPase; Gα: G protein subunit alpha; G_s_: G protein subunit s; β, γ: G protein beta-gamma complex.

To conclude, we provide evidence for the functional role of TRPM4 and TRPM5 in immortalized human parietal cells, wherein both channels are necessary for proton secretion upon stimulation with food-derived bitter compounds by mediating Na^+^ influx. Specifically, our results indicate that HGT-1 cells possess a signal transduction pathway for bitter compounds, which is equal to taste cells, thereby rendering HGT-1 cells a suitable surrogate cell model for investigating the gustatory impact of taste-active compounds. As summarized in Figure 9, the taste signaling pathway partially interacts with the well-known signaling pathway of the secretagogue acetylcholine. Although the precise mode of action and a potential interaction with the cAMP pathway remain to be clarified, this finding may help develop novel approaches that may contribute to treating pathophysiological conditions due to increased gastric acid secretion.

## Data Availability

The data sets generated during and/or analyzed during the current study are not publicly available due to the institutional statutes but are available from the corresponding author on reasonable request.

## Supporting Information Available

LA–ICP–MS parameters (Table S1), cell viability (Figure S1), immunostaining (Figure S2), intracellular Ca^2+^ measurements (Figure S3), intracellular Na^+^ measurements (Figure S4), knockdown efficiency (Figure S5), gene regulation (Figure S6), and pharmacological inhibition (Figure S7) (PDF)

## References

[ref1] Schubert M. L.; Peura D. A. Control of gastric acid secretion in health and disease. Gastroenterology 2008, 134 (7), 1842–1860. 10.1053/j.gastro.2008.05.021. 18474247

[ref2] Yao X.; Forte J. G. Cell biology of acid secretion by the parietal cell. Annu. Rev. Physiol. 2003, 65, 103–131. 10.1146/annurev.physiol.65.072302.114200. 12500969

[ref3] Wess J. Molecular biology of muscarinic acetylcholine receptors. Crit. Rev. Neurobiol. 1996, 10 (1), 69–99. 10.1615/CritRevNeurobiol.v10.i1.40. 8853955

[ref4] Li Z. Q.; Mårdh S. Interactions between Ca^2+^- and cAMP-dependent stimulatory pathways in parietal cells. Biochim. Biophys. Acta 1996, 1311 (2), 133–142. 10.1016/0167-4889(96)00006-7. 8630331

[ref5] Liszt K. I.; Ley J. P.; Lieder B.; Behrens M.; Stöger V.; Reiner A.; Hochkogler C. M.; Köck E.; Marchiori A.; Hans J.; et al. Caffeine induces gastric acid secretion via bitter taste signaling in gastric parietal cells. Proc. Natl. Acad. Sci. U. S. A. 2017, 114 (30), E6260–E6269 10.1073/pnas.1703728114. 28696284 PMC5544304

[ref6] Prawitt D.; Monteilh-Zoller M. K.; Brixel L.; Spangenberg C.; Zabel B.; Fleig A.; Penner R. TRPM5 is a transient Ca^2+^-activated cation channel responding to rapid changes in [Ca^2+^]i. Proc. Natl. Acad. Sci. U. S. A. 2003, 100 (25), 15166–15171. 10.1073/pnas.2334624100. 14634208 PMC299937

[ref7] Liu D.; Liman E. R. Intracellular Ca^2+^ and the phospholipid PIP2 regulate the taste transduction ion channel TRPM5. Proc. Natl. Acad. Sci. U. S. A. 2003, 100 (25), 15160–15165. 10.1073/pnas.2334159100. 14657398 PMC299934

[ref8] Dutta Banik D.; Martin L. E.; Freichel M.; Torregrossa A. M.; Medler K. F. TRPM4 and TRPM5 are both required for normal signaling in taste receptor cells. Proc. Natl. Acad. Sci. U. S. A. 2018, 115 (4), E772–e781 10.1073/pnas.1718802115. 29311301 PMC5789955

[ref9] Margolskee R. F. Molecular mechanisms of bitter and sweet taste transduction. J. Biol. Chem. 2002, 277 (1), 1–4. 10.1074/jbc.R100054200. 11696554

[ref10] Roper S. D. Signal transduction and information processing in mammalian taste buds. Pflugers Arch. - Eur. J. Physiol. 2007, 454 (5), 759–776. 10.1007/s00424-007-0247-x. 17468883 PMC3723147

[ref11] Clapham D. E. TRP channels as cellular sensors. Nature 2003, 426 (6966), 517–524. 10.1038/nature02196. 14654832

[ref12] Hofmann T.; Chubanov V.; Gudermann T.; Montell C. TRPM5 is a voltage-modulated and Ca(2+)-activated monovalent selective cation channel. Curr. Biol. 2003, 13 (13), 1153–1158. 10.1016/S0960-9822(03)00431-7. 12842017

[ref13] Launay P.; Fleig A.; Perraud A. L.; Scharenberg A. M.; Penner R.; Kinet J. P. TRPM4 is a Ca^2+^-activated nonselective cation channel mediating cell membrane depolarization. Cell 2002, 109 (3), 397–407. 10.1016/S0092-8674(02)00719-5. 12015988

[ref14] Ullrich N. D.; Voets T.; Prenen J.; Vennekens R.; Talavera K.; Droogmans G.; Nilius B. Comparison of functional properties of the Ca^2+^-activated cation channels TRPM4 and TRPM5 from mice. Cell Calcium 2005, 37 (3), 267–278. 10.1016/j.ceca.2004.11.001. 15670874

[ref15] Fonfria E.; Murdock P. R.; Cusdin F. S.; Benham C. D.; Kelsell R. E.; McNulty S. Tissue distribution profiles of the human TRPM cation channel family. J. Recept. Signal Transduction Res. 2006, 26 (3), 159–178. 10.1080/10799890600637506. 16777713

[ref16] Kaske S.; Krasteva G.; König P.; Kummer W.; Hofmann T.; Gudermann T.; Chubanov V. TRPM5, a taste-signaling transient receptor potential ion-channel, is a ubiquitous signaling component in chemosensory cells. BMC Neurosci. 2007, 8 (1), 49 10.1186/1471-2202-8-49. 17610722 PMC1931605

[ref17] Laboisse C. L.; Augeron C.; Couturier-Turpin M. H.; Gespach C.; Cheret A. M.; Potet F. Characterization of a newly established human gastric cancer cell line HGT-1 bearing histamine H2-receptors. Cancer Res. 1982, 42 (4), 1541–1548. 6277484

[ref18] Carmosino M.; Procino G.; Casavola V.; Svelto M.; Valenti G. The cultured human gastric cells HGT-1 express the principal transporters involved in acid secretion. Pflugers Arch. - Eur. J. Physiol. 2000, 440 (6), 871–880. 10.1007/s004240000363. 11041553

[ref19] Zopun M.; Liszt K. I.; Stoeger V.; Behrens M.; Redel U.; Ley J. P.; Hans J.; Somoza V. Human Sweet Receptor T1R3 is Functional in Human Gastric Parietal Tumor Cells (HGT-1) and Modulates Cyclamate and Acesulfame K-Induced Mechanisms of Gastric Acid Secretion. J. Agric. Food Chem. 2018, 66 (19), 4842–4852. 10.1021/acs.jafc.8b00658. 29665689

[ref20] Ley J. P.; Krammer G.; Reinders G.; Gatfield I. L.; Bertram H. J. Evaluation of bitter masking flavanones from Herba Santa (*Eriodictyon californicum* (H. and A.) Torr., Hydrophyllaceae). J. Agric. Food Chem. 2005, 53 (15), 6061–6066. 10.1021/jf0505170. 16028996

[ref21] Liszt K. I.; Walker J.; Somoza V. Identification of organic acids in wine that stimulate mechanisms of gastric acid secretion. J. Agric. Food Chem. 2012, 60 (28), 7022–7030. 10.1021/jf301941u. 22708700

[ref22] Andersen G.; Kahlenberg K.; Krautwurst D.; Somoza V. [6]-Gingerol Facilitates CXCL8 Secretion and ROS Production in Primary Human Neutrophils by Targeting the TRPV1 Channel. Mol. Nutr. Food Res. 2023, 67 (4), e2200434 10.1002/mnfr.202200434. 36564924

[ref23] Tiroch J.; Sterneder S.; Di Pizio A.; Lieder B.; Hoelz K.; Holik A. K.; Pignitter M.; Behrens M.; Somoza M.; Ley J. P.; et al. Bitter Sensing TAS2R50 Mediates the trans-Resveratrol-Induced Anti-inflammatory Effect on Interleukin 6 Release in HGF-1 Cells in Culture. J. Agric. Food Chem. 2021, 69 (45), 13339–13349. 10.1021/acs.jafc.0c07058. 33461297

[ref24] Walker J.; Hell J.; Liszt K. I.; Dresel M.; Pignitter M.; Hofmann T.; Somoza V. Identification of beer bitter acids regulating mechanisms of gastric acid secretion. J. Agric. Food Chem. 2012, 60 (6), 1405–1412. 10.1021/jf204306z. 22313115

[ref25] Richter P.; Sebald K.; Fischer K.; Behrens M.; Schnieke A.; Somoza V. Bitter Peptides YFYPEL, VAPFPEVF, and YQEPVLGPVRGPFPIIV, Released during Gastric Digestion of Casein, Stimulate Mechanisms of Gastric Acid Secretion via Bitter Taste Receptors TAS2R16 and TAS2R38. J. Agric. Food Chem. 2022, 70 (37), 11591–11602. 10.1021/acs.jafc.2c05228. 36054030 PMC9501810

[ref26] Meyerhof W.; Batram C.; Kuhn C.; Brockhoff A.; Chudoba E.; Bufe B.; Appendino G.; Behrens M. The molecular receptive ranges of human TAS2R bitter taste receptors. Chem. Senses 2010, 35 (2), 157–170. 10.1093/chemse/bjp092. 20022913

[ref27] Stoeger V.; Liszt K. I.; Lieder B.; Wendelin M.; Zopun M.; Hans J.; Ley J. P.; Krammer G. E.; Somoza V. Identification of Bitter-Taste Intensity and Molecular Weight as Amino Acid Determinants for the Stimulating Mechanisms of Gastric Acid Secretion in Human Parietal Cells in Culture. J. Agric. Food Chem. 2018, 66 (26), 6762–6771. 10.1021/acs.jafc.8b01802. 29879844

[ref28] Stoeger V.; Holik A. K.; Hölz K.; Dingjan T.; Hans J.; Ley J. P.; Krammer G. E.; Niv M. Y.; Somoza M. M.; Somoza V. Bitter-Tasting Amino Acids l-Arginine and l-Isoleucine Differentially Regulate Proton Secretion via T2R1 Signaling in Human Parietal Cells in Culture. J. Agric. Food Chem. 2020, 68 (11), 3434–3444. 10.1021/acs.jafc.9b06285. 31891507

[ref29] Muschol M.; Dasgupta B. R.; Salzberg B. M. Caffeine interaction with fluorescent calcium indicator dyes. Biophys. J. 1999, 77 (1), 577–586. 10.1016/S0006-3495(99)76914-6. 10388782 PMC1300354

[ref30] Engevik A. C.; Kaji I.; Goldenring J. R. The Physiology of the Gastric Parietal Cell. Physiol. Rev. 2020, 100 (2), 573–602. 10.1152/physrev.00016.2019. 31670611 PMC7327232

[ref31] Ley J. P.; Dessoy M.; Paetz S.; Blings M.; Hoffmann-Lücke P.; Reichelt K. V.; Krammer G. E.; Pienkny S.; Brandt W.; Wessjohann L. Identification of enterodiol as a masker for caffeine bitterness by using a pharmacophore model based on structural analogues of homoeriodictyol. J. Agric. Food Chem. 2012, 60 (25), 6303–6311. 10.1021/jf301335z. 22670770

[ref32] Gees M.; Alpizar Y. A.; Luyten T.; Parys J. B.; Nilius B.; Bultynck G.; Voets T.; Talavera K. Differential effects of bitter compounds on the taste transduction channels TRPM5 and IP3 receptor type 3. Chem. Senses 2014, 39 (4), 295–311. 10.1093/chemse/bjt115. 24452633

[ref33] Palmer R. K.; Atwal K.; Bakaj I.; Carlucci-Derbyshire S.; Buber M. T.; Cerne R.; Cortés R. Y.; Devantier H. R.; Jorgensen V.; Pawlyk A.; et al. Triphenylphosphine oxide is a potent and selective inhibitor of the transient receptor potential melastatin-5 ion channel. Assay Drug Dev. Technol. 2010, 8 (6), 703–713. 10.1089/adt.2010.0334. 21158685

[ref34] Grand T.; Demion M.; Norez C.; Mettey Y.; Launay P.; Becq F.; Bois P.; Guinamard R. 9-phenanthrol inhibits human TRPM4 but not TRPM5 cationic channels. Br. J. Pharmacol. 2008, 153 (8), 1697–1705. 10.1038/bjp.2008.38. 18297105 PMC2438271

[ref35] Dell’acqua M. L.; Carroll R. C.; Peralta E. G. Transfected m2 muscarinic acetylcholine receptors couple to G alpha i2 and G alpha i3 in Chinese hamster ovary cells. Activation and desensitization of the phospholipase C signaling pathway. J. Biol. Chem. 1993, 268 (8), 5676–5685. 10.1016/S0021-9258(18)53372-X. 8449930

[ref36] Ghosh D.; Segal A.; Voets T. Distinct modes of perimembrane TRP channel turnover revealed by TIR-FRAP. Sci. Rep. 2014, 4 (1), 7111 10.1038/srep07111. 25407951 PMC4236744

[ref37] Crnich R.; Amberg G. C.; Leo M. D.; Gonzales A. L.; Tamkun M. M.; Jaggar J. H.; Earley S. Vasoconstriction resulting from dynamic membrane trafficking of TRPM4 in vascular smooth muscle cells. Am. J. Physiol. Cell Physiol. 2010, 299 (3), C682–C694. 10.1152/ajpcell.00101.2010. 20610768 PMC2944317

[ref38] Kruse M.; Schulze-Bahr E.; Corfield V.; Beckmann A.; Stallmeyer B.; Kurtbay G.; Ohmert I.; Schulze-Bahr E.; Brink P.; Pongs O. Impaired endocytosis of the ion channel TRPM4 is associated with human progressive familial heart block type I. J. Clin. Invest. 2009, 119 (9), 2737–2744. 10.1172/JCI38292. 19726882 PMC2735920

[ref39] Stokl̷osa P.; Kappel S.; Peinelt C. A Novel Role of the TRPM4 Ion Channel in Exocytosis. Cells 2022, 11 (11), 1793 10.3390/cells11111793. 35681487 PMC9180413

[ref40] Izawa K.; Amino Y.; Kohmura M.; Ueda Y.; Kuroda M. 4.16 - Human–Environment Interactions – Taste. In Comprehensive Natural Products II; Liu H.-W.; Mander L., Eds.; Elsevier: Oxford, 2010; pp. 631 671.

[ref41] Guinard J.-X.; Hong D. Y.; Zoumas-Morse C.; Budwig C.; Russell G. F. Chemoreception and perception of the bitterness of isohumulones. Physiol. Behav. 1994, 56 (6), 1257–1263. 10.1016/0031-9384(94)90374-3. 7878099

[ref42] Sonntag T.; Kunert C.; Dunkel A.; Hofmann T. Sensory-Guided Identification of N -(1-Methyl-4-oxoimidazolidin-2-ylidene)-α-amino Acids as Contributors to the Thick-Sour and Mouth-Drying Orosensation of Stewed Beef Juice. J. Agric. Food Chem. 2010, 58 (10), 6341–6350. 10.1021/jf100591c. 20420435

[ref43] Negulescu P. A.; Harootunian A.; Tsien R. Y.; Machen T. E. Fluorescence measurements of cytosolic free Na concentration, influx and efflux in gastric cells. Cell Regul. 1990, 1 (3), 259–268. 10.1091/mbc.1.3.259. 1712635 PMC361467

[ref44] Roland W. S. U.; van Buren L.; Gruppen H.; Driesse M.; Gouka R. J.; Smit G.; Vincken J. P. Bitter taste receptor activation by flavonoids and isoflavonoids: Modeled structural requirements for activation of hTAS2R14 and hTAS2R39. J. Agric. Food Chem. 2013, 61 (44), 10454–10466. 10.1021/jf403387p. 24117141

[ref45] Huang Y. A.; Roper S. D. Intracellular Ca(2+) and TRPM5-mediated membrane depolarization produce ATP secretion from taste receptor cells. J. Physiol. 2010, 588 (Pt 13), 2343–2350. 10.1113/jphysiol.2010.191106. 20498227 PMC2915511

[ref46] Bigiani A. Does ENaC Work as Sodium Taste Receptor in Humans?. Nutrients 2020, 12 (4), 1195 10.3390/nu12041195. 32344597 PMC7230849

[ref47] Chen M. C.; Wu S. V.; Reeve J. R. Jr.; Rozengurt E. Bitter stimuli induce Ca^2+^ signaling and CCK release in enteroendocrine STC-1 cells: Role of L-type voltage-sensitive Ca^2+^ channels. Am. J. Physiol. Cell Physiol. 2006, 291 (4), C726–C739. 10.1152/ajpcell.00003.2006. 16707556

[ref48] Shah B. P.; Liu P.; Yu T.; Hansen D. R.; Gilbertson T. A. TRPM5 is critical for linoleic acid-induced CCK secretion from the enteroendocrine cell line, STC-1. Am. J. Physiol. Cell Physiol. 2012, 302 (1), C210–C219. 10.1152/ajpcell.00209.2011. 21998136 PMC3328913

[ref49] Brixel L. R.; Monteilh-Zoller M. K.; Ingenbrandt C. S.; Fleig A.; Penner R.; Enklaar T.; Zabel B. U.; Prawitt D. TRPM5 regulates glucose-stimulated insulin secretion. Pflugers Arch. - Eur. J. Physiol. 2010, 460 (1), 69–76. 10.1007/s00424-010-0835-z. 20393858 PMC5663632

[ref50] Krishnan K.; Ma Z.; Björklund A.; Islam M. S. Role of transient receptor potential melastatin-like subtype 5 channel in insulin secretion from rat β-cells. Pancreas 2014, 43 (4), 597–604. 10.1097/MPA.0000000000000027. 24632551

[ref51] Cheng H.; Beck A.; Launay P.; Gross S. A.; Stokes A. J.; Kinet J. P.; Fleig A.; Penner R. TRPM4 controls insulin secretion in pancreatic β-cells. Cell Calcium 2007, 41 (1), 51–61. 10.1016/j.ceca.2006.04.032. 16806463 PMC5663640

[ref52] Pi M.; Wu Y.; Lenchik N. I.; Gerling I.; Quarles L. D. GPRC6A mediates the effects of L-arginine on insulin secretion in mouse pancreatic islets. Endocrinology 2012, 153 (10), 4608–4615. 10.1210/en.2012-1301. 22872579 PMC3512028

[ref53] Philippaert K.; Pironet A.; Mesuere M.; Sones W.; Vermeiren L.; Kerselaers S.; Pinto S.; Segal A.; Antoine N.; Gysemans C.; et al. Steviol glycosides enhance pancreatic beta-cell function and taste sensation by potentiation of TRPM5 channel activity. Nat. Commun. 2017, 8 (1), 14733 10.1038/ncomms14733. 28361903 PMC5380970

[ref54] Pei K.; Ou J.; Huang J.; Ou S. p-Coumaric acid and its conjugates: Dietary sources, pharmacokinetic properties and biological activities. J. Sci. Food Agric. 2016, 96 (9), 2952–2962. 10.1002/jsfa.7578. 26692250

[ref55] Soares S.; Silva M. S.; García-Estevez I.; Groβmann P.; Brás N.; Brandão E.; Mateus N.; de Freitas V.; Behrens M.; Meyerhof W. Human Bitter Taste Receptors Are Activated by Different Classes of Polyphenols. J. Agric. Food Chem. 2018, 66 (33), 8814–8823. 10.1021/acs.jafc.8b03569. 30056706

[ref56] Yamashita Y.; Sakakibara H.; Toda T.; Ashida H. Insights into the potential benefits of black soybean (Glycine max L.) polyphenols in lifestyle diseases. Food Funct. 2020, 11 (9), 7321–7339. 10.1039/D0FO01092H. 32852022

[ref57] Xing L.; Zhang H.; Qi R.; Tsao R.; Mine Y. Recent Advances in the Understanding of the Health Benefits and Molecular Mechanisms Associated with Green Tea Polyphenols. J. Agric. Food Chem. 2019, 67 (4), 1029–1043. 10.1021/acs.jafc.8b06146. 30653316

