# Supplementary material for: Sodium-Permeable Ion Channels TRPM4 and TRPM5 are Functional in Human Gastric Parietal Cells in Culture and Modulate the Cellular Response to Bitter-Tasting Food Constituents
Source: J Agric Food Chem. 2024 Feb 20;72(9):4906–17. doi: 10.1021/acs.jafc.3c09085 (PMC10921469; doi:10.1021/acs.jafc.3c09085)
Supplement: Supplementary file 1 — jf3c09085_si_001.pdf [file jf3c09085_si_001.pdf]

Supporting information to

Sodium Permeable Ion Channels TRPM4 and TRPM5 are Functional in Human Gastric Parietal Cells in Culture and Modulate the Cellular Response to Bitter Tasting Food Constituents

By

P. Richter[1,2#], G. Andersen[2#], K. Kahlenberg[2], A. U. Mueller[2], P. Pirkwieser[2], V. Boger[2], V. Somoza[2,3,4*]

[1]TUM School of Life Sciences Weihenstephan, Technical University of Munich, Alte Akademie 8, 85354 Freising, Germany

[2]Leibniz Institute for Food Systems Biology at the Technical University of Munich, Lise-Meitner-Str. 34, 85354 Freising, Germany

[3]Chair of Nutritional Systems Biology, TUM School of Life Sciences, Technical University of Munich, Lise-Meitner-Str. 34, 85354 Freising, Germany

[4]Department of Physiological Chemistry, Faculty of Chemistry, University of Vienna, Josef-Holaubek-Platz 2 (UZA II), 1090 Wien, Austria

[#]The authors contributed equally to this work

**Correspondence to Veronika Somoza: telephone +49-8161-71-2700, E-mail: v.somoza.leibniz-lsb@tum.de*

Table S1: Instrumental parameters for the LA-ICP-MS measurements.

NexION 5000 Multi-Quadrupole ICP-MS		Iridia 193 nm Laser Ablation System	
**Software**	Syngistix v.3.3	**Software**	Chromium v.3.1
**Nebulizer Gas Flow [L/min]**	0.82-0.94	**Data Processing Software**	HDIP-v1.7.1
**Auxiliary Gas Flow [L/min]**	1.20	**Ablation Cell Type**	Cobalt Long Pulse with Z-prime (100 mm x 100 mm)
**Plasma Gas Flow [L/min]**	16	**Fluence [J/cm^2]**	1.0
**ICP RF Power [W]**	1600	**Repetition Rate [Hz]**	162
**Profile**	standard	**Spot size [µm]**	3
**Analyte**	Na	**Mask shape**	circle
**Scan Mode**	MS/MS	**Image xy dimensions [µm]**	200x175
**Q1/Q3 Begin Mass**	22.9898	**Lateral scan speed [µm/s]**	52
**IGM**	focusing	**Dosage**	9
**Dwell Time Per AMU**	50	**Scan mode**	line by line uni-directional, laser off between rows
**Rpq [V]**	0.25	**Image xy dimensions [µm]**	200x175

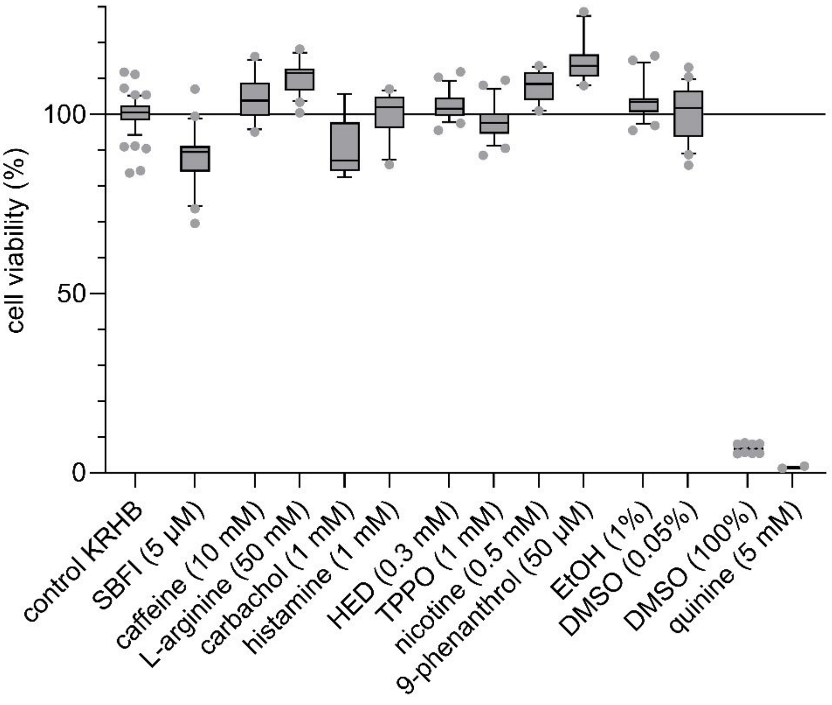

Figure S1: Results of the experiments on cell viability. No relevant effects of viability after incubation of the investigated substances were found. DMSO (100%) and quinine (5 mM) were used as negative controls. Data were normalized to HGT-1 cells treated with KRHB only, n = 4-5, t. r. = 3-6.

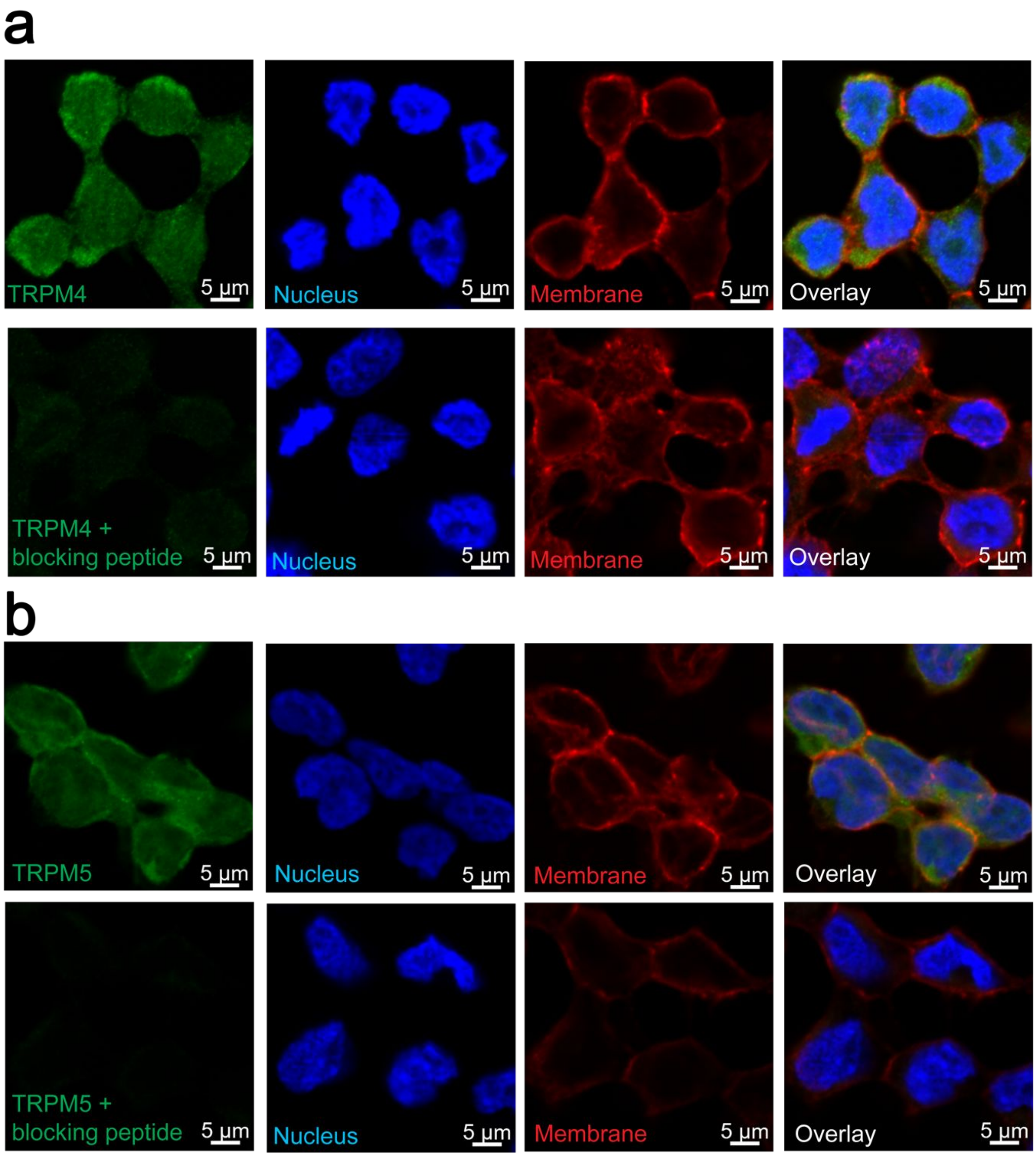

Figure S2: Specific immunostaining of TRPM4 and TRPM5 in HGT-1 cells. Receptor expression is detected by anti-TRPM4 antibody (a) and anti-TRPM5 antibody (b) in combination with an Alexa Fluor 488 anti-rabbit IgG (green). The nucleus is visualized by Hoechst-33342 (blue), and the plasma membrane by biotin-conjugated concanavalin A binding in combination with Alexa Fluor 633 conjugate (red). As antibody specificity control, primary antibodies were preincubated with the corresponding blocking peptide. Scale: 5 µM.

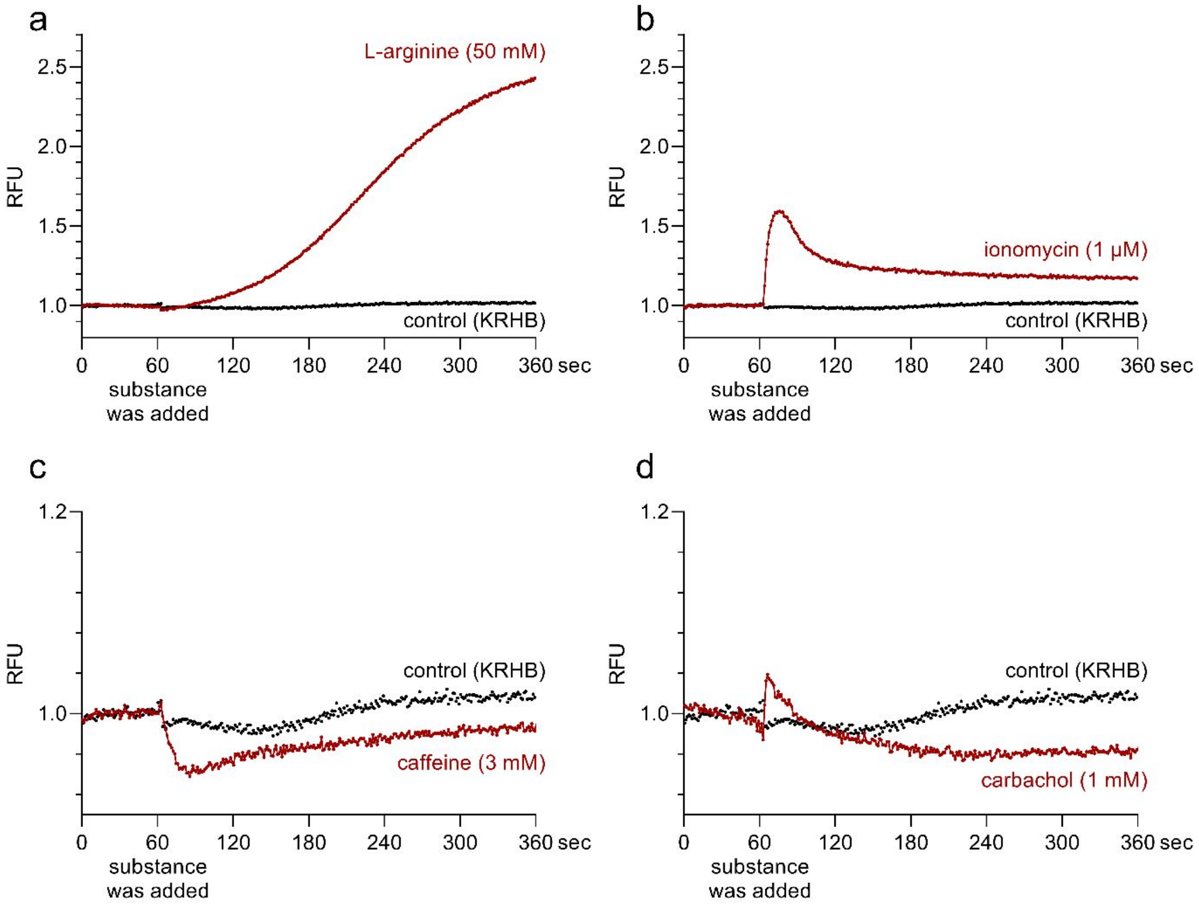

Figure S3: L-arginine (50 mM, a) and carbachol (1 mM, d) lead to an increase in intracellular Ca^{2+} concentration in HGT-1 cells, whereas this is not measurable for caffeine due to its already described quenching effect (3 mM, c). Ionomycin (1 µM, b) was used as a positive control. Substances were added after 60 seconds. In comparison, the untreated control (black) is shown. Data are shown as mean, n = 4, t. r. = 2.

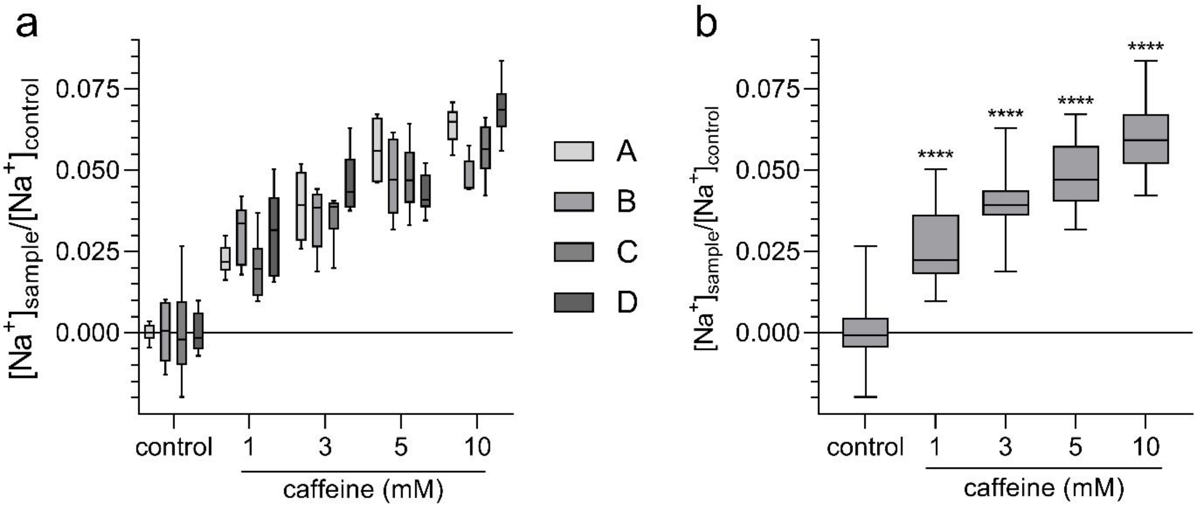

Figure S4: Caffeine induced increases in intracellular Na^+ are stable and reproducible. (a) Each grayscale represents the results of one biological replicate (t. r. = 6). In all cases, a reproducible increase in sodium concentrations in HGT-1 cells was shown starting at concentrations of 1 mM caffeine. (b) The combined results of the four biological replicates. Statistics: n = 4, t. r. = 6, one-way ANOVA Holm-Šidák *post hoc* test; significant differences are expressed with **** = $p \leq 0.0001$.

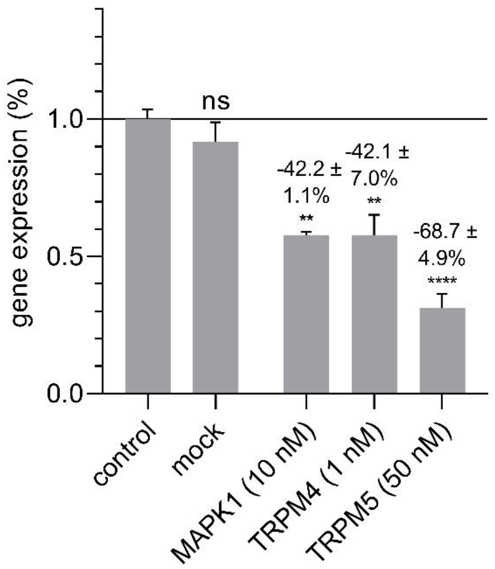

Figure S5: Gene expression of TRPM4 and TRPM5 were reduced by incubating HGT-1 cells with lipofectamine and siRNA for 72 hours. The expression of MAPK1 was downregulated as a positive control. Nonspecific siRNA was used as a negative control (mock). Knock-down efficiencies are shown as mean ± SEM, n = 4, t. r. = 3, statistics: one-way ANOVA Holm-Šidák *post hoc* test; significant differences are expressed with ** = $p \leq 0.01$, **** = $p \leq 0.0001$.

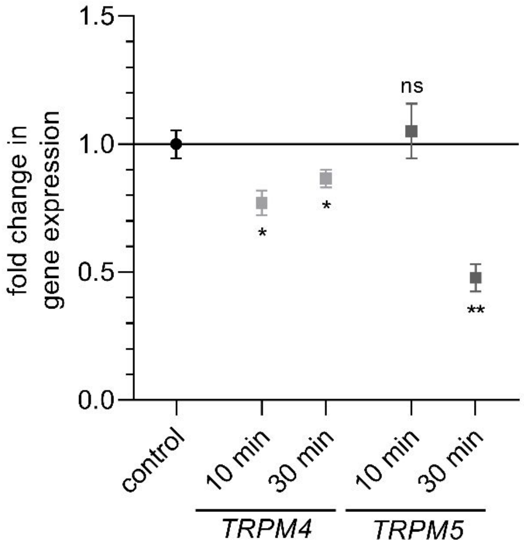

Figure S6: Incubation of the cells with caffeine (3 mM) resulted in a significant down-regulation of TRPM4 at the RNA level after only 10 minutes. This regulation was also still present after 30 minutes. For the expression of TRPM5, there was no significant change after 10 minutes, but after 30 minutes the expression was strongly downregulated. Data are shown as mean ± SEM, n = 4, t. r. = 3, statistics: one-way ANOVA Holm-Šidák *post hoc* test; significant differences are expressed with * = $p \leq 0.05$, ** = $p \leq 0.01$.

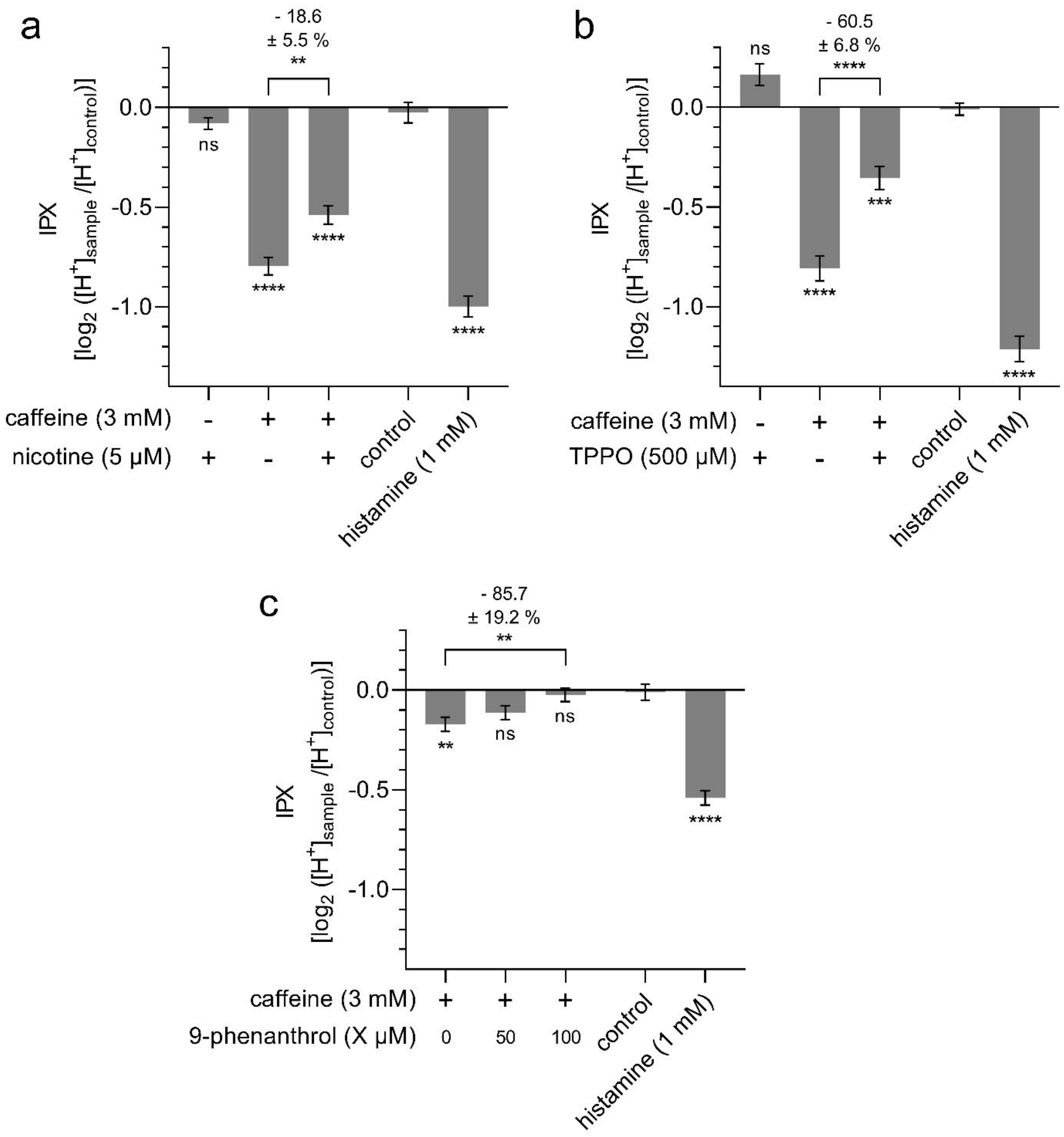

Figure S7: Pharmacological inhibition with the TRPM5-specific inhibitors (a) nicotine and (b) TPPO and the TRPM4-specific inhibitor (c) 9-phenanthrol resulted in a reduction in caffeine-induced (3 mM) stimulation of proton secretion of HGT-1 cells. Data are shown as mean ± SEM, n = 4, t. r. = 6, statistics: one-way ANOVA Holm-Šidák *post hoc* test; significant differences are expressed with ** = $p \leq 0.01$, *** = $p \leq 0.001$, **** = $p \leq 0.0001$.
